# Dopamine Dynamics and Neurobiology of Non-Response to Antipsychotics, Relevance for Treatment Resistant Schizophrenia: A Systematic Review and Critical Appraisal

**DOI:** 10.3390/biomedicines11030895

**Published:** 2023-03-14

**Authors:** Felice Iasevoli, Camilla Avagliano, Luigi D’Ambrosio, Annarita Barone, Mariateresa Ciccarelli, Giuseppe De Simone, Benedetta Mazza, Licia Vellucci, Andrea de Bartolomeis

**Affiliations:** Laboratory of Molecular and Translational Psychiatry, Unit of Treatment Resistant Psychosis, Section of Psychiatry, Department of Neuroscience, Reproductive Science and Odontostomatology, University of Naples Federico II, 80131 Napoli, Italy; felice.iasevoli@unina.it (F.I.);

**Keywords:** psychosis, clozapine, refractory, positive symptoms, negative symptoms, glutamate, ultra-resistant

## Abstract

Treatment resistant schizophrenia (TRS) is characterized by a lack of, or suboptimal response to, antipsychotic agents. The biological underpinnings of this clinical condition are still scarcely understood. Since all antipsychotics block dopamine D2 receptors (D2R), dopamine-related mechanisms should be considered the main candidates in the neurobiology of antipsychotic non-response, although other neurotransmitter systems play a role. The aims of this review are: (i) to recapitulate and critically appraise the relevant literature on dopamine-related mechanisms of TRS; (ii) to discuss the methodological limitations of the studies so far conducted and delineate a theoretical framework on dopamine mechanisms of TRS; and (iii) to highlight future perspectives of research and unmet needs. Dopamine-related neurobiological mechanisms of TRS may be multiple and putatively subdivided into three biological points: (1) D2R-related, including increased D2R levels; increased density of D2Rs in the high-affinity state; aberrant D2R dimer or heteromer formation; imbalance between D2R short and long variants; extrastriatal D2Rs; (2) presynaptic dopamine, including low or normal dopamine synthesis and/or release compared to responder patients; and (3) exaggerated postsynaptic D2R-mediated neurotransmission. Future points to be addressed are: (i) a more neurobiologically-oriented phenotypic categorization of TRS; (ii) implementation of neurobiological studies by directly comparing treatment resistant vs. treatment responder patients; (iii) development of a reliable animal model of non-response to antipsychotics.

## 1. Introduction

Schizophrenia is among the most debilitating disorders in psychiatry, with great impairment of social and individual functioning [1]. Although antipsychotics are the cornerstone of schizophrenia treatment, 20–45% of patients show partial or no response to antipsychotic medications [2]. According to the American Psychiatric Association (APA) practice guidelines [3], treatment resistant schizophrenia (TRS) may be defined as “little or no symptomatic response to multiple (at least two) antipsychotic trials of adequate duration (at least 6 weeks) and dose (therapeutic range)”. In agreement with this definition, poor response to antipsychotics may have a clinical, pharmacokinetic, or pharmacodynamic origin, the latter being the only mechanism assumed to induce a state of “true” treatment resistance, which is due to non-modifiable, drug-specific factors.

Schizophrenia treatment may be completely or partially unsuccessful for multiple clinical reasons, including (but not limited to) the possibility that patients may be receiving a suboptimal dose of antipsychotics, may suffer from comorbid substance misuse, or may be under concurrent use of other prescribed medicines or a concomitant physical illness that may negatively impact antipsychotic treatment. Genetic or iatrogenic variations in antipsychotic pharmacokinetics may also lead to inadequate blood levels of the drug and ineffective drug concentrations at the site of action [4,5,6,7]. These causes of non-response to antipsychotics should always be investigated and ruled out before considering putative pharmacodynamic mechanisms of resistance.

In a substantial number of non-responder patients, however, clinical variables can be reasonably excluded, and no pharmacokinetics variations can be suspected. These patients may putatively represent treatment resistant cases due to pharmacodynamic causes [6]. Nonetheless, despite the clinical relevance of this phenomenon, biological underpinnings of pharmacodynamic-related mechanisms of non-response to antipsychotics, and more in general of TRS, are still scarcely understood [8].

Schizophrenia has been prominently, albeit not exclusively, regarded as a dysfunction of central dopamine neurotransmission [9,10,11]. From a neurobiological perspective, schizophrenia is considered a neurodevelopmental disorder with complex genetic architecture and pathophysiology [12]. Multiple genetic loci have been associated to schizophrenia, although the precise pathophysiological function of several genetic elements is yet to be determined [13]. However, schizophrenia has been considered a disorder of the transcriptome, and aberrant microRNAs may operate to affect the expression of genes implicated in neurodevelopment and in specific neurotransmitter signaling [14]. Among the more studied and corroborated pathophysiological hypotheses of schizophrenia are the dopaminergic dysregulation and the disturbed glutamatergic neurotransmission hypotheses [15]. The dopamine dysregulation hypothesis postulates that the presynaptic compartment is the major site of dopaminergic dysfunction and, specifically, elevated dopamine synthesis and release capacity [15]. However, several studies have also explored putative dysfunctions of D2 receptor levels at both pre and postsynaptic sites, as well as disturbance of D2-related postreceptor signaling [15]. One putative crucial pathophysiological mechanism, which may also be relevant to TRS, is an imbalance of dopamine–glutamate interplay occurring at the postsynaptic density, which is a protein mesh of the glutamatergic postsynapse devoted to integrating synaptic signaling from different afferent neurons [16].

Remarkably, all antipsychotic drugs share a variable degree of dopamine D2 receptor (D2R) blockade, even if with different affinities and acting as antagonists or partial agonists [17]. More specifically, antipsychotics have been classically subdivided into typical (or neuroleptics) and atypical agents [18]. A more modern classification refers to these agents as first, second, and third-generation antipsychotics. Typical, i.e., first-generation antipsychotics are predicted to be efficacious against positive symptoms but to cause high rates of extrapyramidal side effects (EPS) [18]. Second-generation antipsychotics are demonstrated to be efficacious against positive symptoms but trigger less severe EPS and have partial efficacy against other symptom domains of schizophrenia, namely positive and cognitive symptoms [18]. Despite this schematization, however, differences between antipsychotic agents are several and difficult to encapsulate into schematic representations. Grossly, first-generation antipsychotics share a high affinity and selectivity to D2Rs and block a substantial portion of subcortical D2Rs [19,20]. Second-generation antipsychotics have lower affinity to D2Rs and have less selectivity, with relevant action on 5HT2A, which has been considered one of the pharmacological mechanisms to prevent dopamine loss in the basal ganglia and the occurrence of EPS [20]. Moreover, multiple second-generation antipsychotics have a multireceptor profile, with several neurotransmitter systems targeted and possibly responsible both for therapeutic efficacy and adverse effects [20]. A schematic representation or antipsychotics’ receptor profile in terms of affinity and dissociation constants is given in Table 1 and Table 2, respectively.

Based on these considerations, multiple neurotransmitters have also been implicated in the pathophysiology of schizophrenia [64] and should represent novel targets for future antipsychotic drugs [65]. In addition, structural and functional brain changes have been reported to occur in both first-episode psychosis [66] and multiple-episode chronic schizophrenia patients [67]. However, a clear role of the above-mentioned mechanisms in the pathophysiology of schizophrenia and in the therapeutic action of antipsychotic agents is yet to be fully elucidated.

Despite the possible involvement of other neurotransmitter systems, dopamine dysfunction is still considered the most relevant common pathway leading to schizophrenia [68], and all antipsychotic drugs impact dopamine neurotransmission. Therefore, dopamine-related mechanisms should be considered as one of the main candidates in the neurobiology of non-response to antipsychotics, at least in the main part of patients who develop an acute or progressive loss of response to these agents. According to these points, the aim of this review is to provide an analytical evaluation and a critical appraisal of emerging putative dopamine-related mechanisms of non-response to antipsychotics, which is the base for diagnosing TRS.

## 2. Materials and Methods

**Figure 1:**
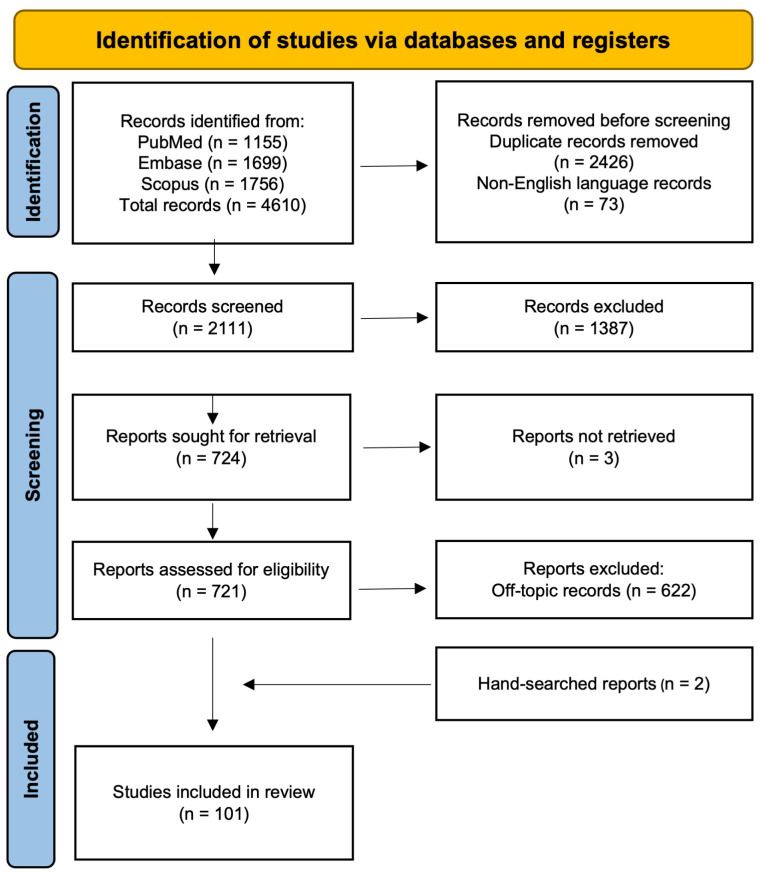
PRISMA diagram showing the flow of information through the different phases of the systematic review.

A comprehensive review of the literature was carried out through Medline/Pubmed, Embase, and Scopus databases. Keywords were searched in the Title/Abstract fields; no date restriction was set; only publications in the English language were included. The Preferred Reporting Items for Systematic Reviews and Meta-Analyses (PRISMA) guidelines [69] were followed in the screening procedure. The search strings included keywords related to treatment resistance/unresponsiveness, schizophrenia/psychotic disorders, and dopamine. The search string is reported as follows: ((((((((((((((“treatment resistant”[Title/Abstract]) OR (treatment-resistant[Title/Abstract])) OR (“ultra resistant”[Title/Abstract])) OR “resistance”[Title/Abstract])) OR (“resistant”[Title/Abstract])) OR (“treatment refractory”[Title/Abstract])) OR (“refractory”[Title/Abstract])) OR (treatment-refractory[Title/Abstract])) OR (“refractoriness”[Title/Abstract])) OR (“unresponsive”[Title/Abstract])) OR (non-responsive[Title/Abstract])) OR (“clozapine”[Title/Abstract])) OR (ultra-resistant[Title/Abstract])) AND (((((“first episode psychosis”[Title/Abstract]) OR (“schizophrenia”[Title/Abstract])) OR (“psychotic”[Title/Abstract])) OR (“psychosis”[Title/Abstract])) OR (“first episode”[Title/Abstract]))) AND (“dopamine”[Title/Abstract]). The latest update of available literature was conducted on 17 February 2023. The search returned 4610 articles. After removing duplicates, 2184 papers were retrieved. All publications were screened by title and abstract to remove not pertinent articles. The outcomes at this stage were: (i) any type of direct neurobiological comparison between treatment resistant and treatment responder patients involving dopamine-related mechanisms; and (ii) any type of dopamine-related neurobiological correlate of response to antipsychotics. After this step, a total of 724 publications were selected, and the full paper of all was read. Again, non-pertinent publications were removed, and the resulting ones formed the literature base for the present work. These latter steps were carried out separately and in blind by two experimenters (L.V., G.D.S.). In case of lack of agreement (publications removed by one and included by the other), the first author was in charge of deciding. Additional publications were hand-searched based on the references of included publications. At the end of the screening process, a total of 101 articles were included in the qualitative synthesis. Figure 1 illustrates the PRISMA flow diagram, showing the different stages of paper selection. Additional reports on dopamine-related molecular mechanisms putatively implicated in the pathophysiology of schizophrenia or in the antipsychotic mechanism of action were included in the manuscript to enlarge the discussion of the purported dopamine basis of resistance to antipsychotic treatment.

## 3. Results

The systematic review of reports yielded 101 studies that met inclusion criteria. Given the large methodological heterogeneity of studies included, ranging from preclinical cell culture paradigms to animal models of non-response to antipsychotics to human neuroimaging reports on dopamine system dysfunctions in treatment resistant schizophrenia patients, we were unable to carry out a quantitative synthesis of evidence.

The lack of a well-defined theoretical framework (at least, in relation to the topic of the present review) in multiple studies also prevented the possibility of proceeding with a hypothesis-driven approach, i.e., reporting different hypotheses to explain dopamine-related underpinnings of TRS and listing studies confirming or rejecting each hypothesis.

In the attempt to summarize the data, we provided a qualitative synthesis of reports, subdividing them into four large putative neurobiological sites of dopamine-related non-response to antipsychotics, namely: 1. dopamine genes related (*n* = 9 reports); 2. D2 receptor dynamics related (*n* = 37 reports); 3. dopamine presynaptic alterations related (*n* = 35 reports); 4. dopamine postsynaptic site alterations related (*n* = 20 reports).

### 3.1. Genetic Variants in Dopaminergic Genes and Response to Antipsychotics

Multiple pharmacogenomic studies have been carried out to associate the response to antipsychotics with specific variants in target genes implicated in dopaminergic signaling. The results, however, are inconclusive to date.

The most replicated studies were based on the evaluation of two genetic loci within the *DRD2* gene, i.e., *TaqI* and *-141C*, whose *A1* and *Del* alleles, respectively, were associated with a reduction of striatal D2Rs [70]. Inconsistent findings were described throughout the studies carried out [70] for several methodological challenges: disparity in populations included differences in antipsychotics investigated and inconsistency in the definition of response to antipsychotics. However, a well-conducted meta-analysis showed a significantly lower response to antipsychotics in *-141C* locus *Del* carriers (both hetero and homozygotes) compared to *Ins/Ins* subjects [71]. This finding was confirmed in sensitivity analyses, although it failed to reach significance when studies including first-episode patients were excluded [71]. In the same meta-analysis, no significant differences in response to antipsychotics were found between *TaqI A1* vs. *A2* allele carriers [71].

The large-scale genome-wide association study from the Psychiatric Genomics Consortium (PGC) has found that the top single-nucleotide polymorphism (SNP), i.e., *rs2514218*, associated with schizophrenia was located about 47 kb upstream of the *DRD2* gene sequence [13]. The *rs2514218* SNP common variant coded for a *C* base, while the polymorphic allele for a *T* base, with *C* carriers, showed a higher risk of schizophrenia [13]. The biological function of this SNP has not been characterized yet, and biological assays from blood and postmortem brain tissues have not identified a relationship between *rs2514218* and *D2R* gene expression levels [13]. Nonetheless, a pharmacogenomic study has reported a significant improvement of positive symptoms in C/C homozygotes first-episode schizophrenia patients treated with aripiprazole or risperidone for 12 weeks compared to *T* carriers under the same conditions [72]. Additionally, *C/C* homozygote patients were more likely to develop akathisia under aripiprazole treatment, while male *T* carriers were more at risk of increased prolactin levels while taking risperidone [72]. The same polymorphism has also been associated with the response to a 6-month treatment with clozapine (Table 3) [73]. The biological mechanisms of these effects are not clear, and it is not to exclude that the *rs2514218* SNP may be merely tagging the effect of other *D2R* gene variants in the antipsychotic response.

A meta-analytic study has demonstrated an association between the catechol-O-methyltransferase (*COMT*) *rs4680* (*Val156Met*) polymorphism and the response to at least atypical antipsychotics in schizophrenia and schizoaffective patients [74]. Specifically, *Met/Met* homozygote patients were more likely to respond and experience greater improvement in positive symptoms compared to *Val* carriers [74]. Other analysis also suggests the possibility that the *COMT rs4680* polymorphism *Val/Val* allele could influence the favorable negative symptom response to clozapine (Table 3) [75]. Notably, *Met* homozygotes have a 3 to 4 times lower enzymatic activity of *COMT* than *Val* homozygotes [76], which translates into a lower COMT-mediated dopamine degradation in cortical regions and, thus, in heightened cortical dopamine transmission. Huang et al. [74] explain the association found in the context of the tonic-phasic dopamine hypothesis [77,78]. They postulate that schizophrenia symptoms and, possibly, the response to antipsychotics may be linked to the differential tonic vs. phasic modality of dopamine release, which in turn, differentially modulates dopamine neurotransmission. However, this hypothesis, at least relative to the assumptions on response to antipsychotics, still needs to receive experimental support.

Notably, a recent genetic association study has reported that the percentage of treatment resistant patients with the *Met* allele of *rs4680* on the *COMT* gene and *C/C* homozygote of *rs3470934* on the glutamate decarboxylase 1 (*GAD1*) gene was significantly higher than in treatment responders and healthy control subjects [79]. The authors speculated that the *Met/CC* allelic combination may predispose to TRS as a consequence of higher dopamine levels and lower γ-Aminobutyric acid (GABA) expression in the prefrontal cortex (PFC), thereby causing an excitation/inhibition imbalance that cannot be reverted by antipsychotics (Table 3).

Other pharmacogenomic studies have been conducted on genetic polymorphisms in *D1R*, *D2R*, *D3R*, *D4R*, or dopamine transporter (*DAT*) genes, whose functional meanings on gene expression or protein sequence are still not characterized and whose neurobiological relevance is yet elusive [70].

Recently, an association study in Mexican schizophrenia patients related the single-nucleotide polymorphism *A-241G* of the *DRD2* gene and the *Met/Met* allele of *COMT* and *Ser/Gly* allele of *DRD3* genes with resistance to treatment (Table 3) [80]. Nonetheless, multiple systematic reviews and meta-analyses have failed to find consistent evidence of high effect size associations [81,82].

As a summary of reports, no firm conclusions can be drawn at the moment from pharmacogenomic studies on dopamine-related neurobiological mechanisms of response/non-response to antipsychotics.

**Table 3:** Comparison of treatment resistant and treatment responder patients on dopamine-related genetic outcomes.

Study Design	Model/ Subjects	Methodology	Main Outcomes	Reference
Preclinical human study	107 TRS (70 males 37 females)	PCR-based restriction fragment length and direct sequencing	COMT rs4680 polymorphism Val/Val allele could influence the favorable negative symptoms’ response to clozapine.	[75]
Prospective human study	208 TRS	Real-time PCR and genotyping	Association between DRD2 rs2514218 and response to clozapine.	[73]
Genetic Association human study	49 TRS 33 UTRS 88 treatment responders	Genotyping	Treatment response could associate with the Val (COMT/ Val158Met) and Ser (DRD3/Ser9Gly) alleles TRS may correlate with the G allele (DRD2/A-241G), UTRS may associate with the Met allele (COMT/Val158Met) and Gly allele from Ser9Gly (DRD3).	[80]
Genetic Association human study	171 TRS 592 treatment responders 447 HC	Genotyping	Rates of treatment resistant patients with the Met allele of rs4680 on the COMT gene and the C/C homozygote of rs3470934 on the GAD1 gene were significantly higher than treatment responders and HC.	[79]

TRS = treatment resistant schizophrenia; PCR = polymerase chain reaction; UTRS = ultra-treatment resistant schizophrenia; COMT = catechol-O-methyltransferase; GAD1 = glutamate decarboxylase 1.

### 3.2. D2R-Related Mechanisms

**Figure 2:**
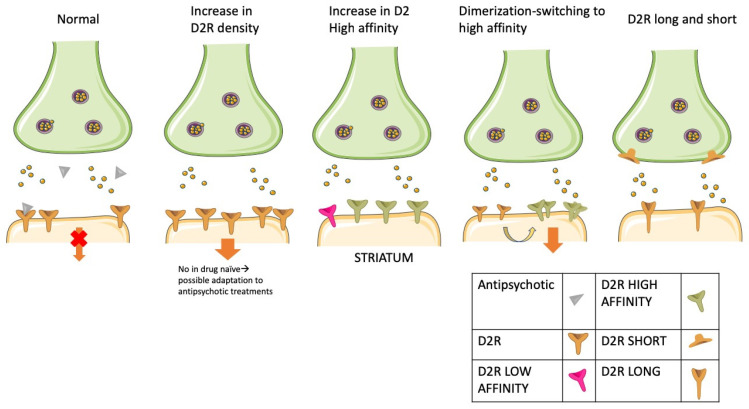
Putative dopamine D2 receptor (D2R)-mediated molecular mechanisms of treatment resistance. The figure illustrates some of the most widely studied putative mechanisms of resistance to antipsychotics associated with D2R dysfunctions. From left to right: (1) dopaminergic synapse in a condition of complete response to antipsychotics. Antipsychotic molecules occupy and block most postsynaptic D2Rs, thus hampering downstream signaling; (2) increase of postsynaptic D2R density. The antipsychotic fails to block a sufficient proportion of D2Rs. Downstream signaling is allowed despite antipsychotic molecules in the synapse. This mechanism has not been observed in drug naïve patients, which may be a consequence of the treatment; (3) a high proportion of receptors is in a high-affinity state, increasing receptor affinity to endogenous dopamine, which competes with and overcomes antipsychotic molecules to bind D2Rs. As a result, antipsychotics fail in antagonizing the D2R-mediated downstream signaling pathway; (4) D2R homodimerization, which fosters the molecular switch from low to D2R high-affinity state, recapitulating the conditions described in point 3; (5) imbalance between the expression levels of the autoinhibitory presynaptic short D2R isoform (D2Short) and of the postsynaptic long D2R isoform (D2Long). Low expression of D2Short or a ratio shift toward D2Long isoforms causes the inability to stop dopamine overload in the synapse.

A poor or absent response to antipsychotics may theoretically derive from heightened D2R-mediated transmission that conventional D2R blocking agents do not revert. In turn, multiple mechanisms have been accounted for: increased D2R levels; increased density of D2Rs in the high-affinity state; aberrant D2R dimers or heteromers formation; imbalance between D2R short and long variants. In many cases, the same mechanism has accounted for both schizophrenia and TRS pathophysiology. According to these views, the difference between schizophrenia and TRS should be quantitative (a larger aberrant D2R-mediated transmission in TRS than in schizophrenia) rather than qualitative. However, only a few of these hypotheses have been tested in human subjects. A graphical summarization of the D2R-related hypothesis for the response to antipsychotics is depicted in Figure 2.

#### 3.2.1. D2R Levels

It has been proposed that schizophrenia patients have higher levels of D2Rs, at least in the striatum, compared to non-psychotic subjects. The elevated D2R levels would explain the supposed hyperdopaminergia, in turn leading to positive psychotic symptoms. Along these lines, non-response to antipsychotics may thus depend on extremely high D2R levels, causing an increased D2R-mediated dopamine transmission that cannot be reverted by conventional antipsychotics.

Despite early data reporting an increase in D2R levels in the striatum of schizophrenia patients compared to controls [83], most subsequent studies failed to replicate this finding in naïve patients [84,85]. Subsequent meta-analyses showed only a moderate, if any, effect size for differences between schizophrenia patients and controls [86,87].

In a later meta-analysis, this modest effect size was lost when including only drug-naïve patients [11], suggesting that a putative increase in D2Rs may represent an adaptation to antipsychotic treatments rather than an inherent pathophysiological feature of schizophrenia. At the moment, no consistent evidence has been provided that D2R levels may be higher in schizophrenia patients compared to controls. Therefore, even the possibility that non-response to antipsychotics may derive from abnormally high D2R levels appears to be erratic.

#### 3.2.2. D2R Low vs. High-Affinity State

One proposed mechanism for psychosis is that the levels of D2Rs in the high-affinity state (rather than the whole D2R pool) are increased during psychotic conditions [88]. According to this hypothesis, D2Rs may exist in two functional states: a high-affinity one (D2^High^), with high affinity for endogenous and exogenous agonists, that is linked to second messenger cascades and a low-affinity one (D2^Low^) that is functionally inert [88]. It has been reported that an up-regulation of D2^High^ represents the final common lesion of all preclinical models of psychosis [89]. Thereby, excessive D2^High^-mediated dopamine neurotransmission may be at the basis of striatal hyperdopaminergia that has been described in schizophrenia [90]. Accordingly, the lack of response to antipsychotics may be caused by extremely up-regulated D2^High^.

However, the actual existence of D2Rs in two affinity states in vivo is still debated and a matter of investigation [91]. Unfortunately, in vivo studies are challenged by the difficulty of developing a selective radioligand for D2^High^ receptors. One available radiotracer is [^11^C]-(+)-PHNO, which is a D2R and D3R agonist reported to bind D2^High^ [56], despite that this point is questioned [92]. Positron emission tomography (PET) studies by [^11^C]-(+)-PHNO failed to find any difference in D2R binding between antipsychotic-naïve patients, clinical-high-risk subjects, and healthy controls, both in resting conditions or under a cognitive task considered increased striatal dopamine release [56,93].

However, [^11^C]-(+)-PHN O is regarded to have a 50-fold higher affinity to D3RS over D2Rs [94], which may possibly hamper selectivity to detect D2^High^. For this reason, in another study, the [^11^C]-I-2-CH3O-N-n-propylnorapomorphine ([^11^C]MNPA) was used as the radioligand, since it has an almost identical affinity to D2RS and D3Rs and may be more suitable to study D2^High^ density [95]. However, again no significant differences in radioligand binding were found in antipsychotic-naïve patients compared to healthy controls [95]. Nonetheless, this study revealed that the ratio of [^11^C]MNPA to [^11^C]raclopride binding in the putamen of schizophrenia patients was higher than in controls [95], putatively reflecting a larger proportion of D2Rs in the high-affinity state in patients compared to controls. At the moment, the possibility that psychosis may depend on heightened levels of D2Rs in the high-affinity state is still a controversial issue.

#### 3.2.3. Dopamine Supersensitivity

Treatment resistant patients may be empirically divided into two groups: (i) those who have not responded to antipsychotics since illness onset, and (ii) those who had responded adequately but experienced a decline in response and psychotic relapses despite stable long-lasting antipsychotic therapy (Table 4) [96,97,98]. Part of these latter patients may be suffering from the so-called dopamine supersensitivity psychosis (DSP) or antipsychotic-induced supersensitivity psychosis [99], which has been related to a compensatory increase in postsynaptic D2R levels or enhanced shift of D2Rs to high-affinity states during long-term antipsychotic treatments [100]. Notably, DSP has been regarded as a pivotal factor in TRS, at least the acquired subtype (Table 4) [98,101].

In this state, schizophrenia patients with at least 1-year under antipsychotic medication (excluding quetiapine and clozapine) and who are compliant to the therapy experience: (1) re-appearance of positive psychotic symptoms despite ongoing adequate antipsychotic therapy; (2) abnormal involuntary movements; (3) absent or negligible life events that can exacerbate the psychosis [102]. Generally, this condition is overcome by an increase in the antipsychotic dose [103], although this strategy is not invariably efficacious. Preclinical studies on animal models comply with observations in humans. Indeed, ongoing treatments with haloperidol and olanzapine progressively lose their efficacy in suppressing amphetamine-induced locomotion and conditioned avoidance responses in rats [104].

**Figure 3:**
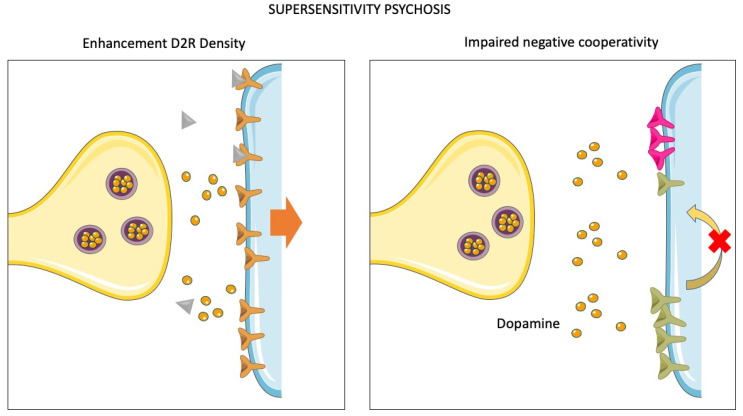
Molecular mechanisms of supersensitivity psychosis. Dopamine supersensitivity psychosis (DSP) is a condition of acquired treatment resistance. An accurate definition requires three clinical features: (1) re-appearance of positive psychotic symptoms despite ongoing antipsychotic therapy; (2) abnormal involuntary movements; (3) absent or negligible life events exacerbating the psychosis. The figure illustrates two different putative mechanisms involved in the pathophysiology of the DSP-acquired form of TRS. **Left panel:** chronic or subchronic exposure to antipsychotics yields an adaptation of postsynaptic sites, which increase D2R expression on the membrane to overcome receptor blockade and diminish D2R-mediated downstream signaling. **Right panel:** under physiological circumstances, D2Rs can aggregate in oligomers. The binding of dopamine to one D2R causes, in turn, the unoccupied receptors to switch to a low-affinity state. The disruption of this mechanism of “negative cooperativity” is supposed to cause a condition of supersensitivity to dopamine. Indeed, antipsychotics would no longer be able to block downstream signaling.

One theoretical explanation for acquired dopaminergic supersensitivity could be an increase in D2R density after long-term treatment with antipsychotics [105], consistent with the view that the treatment resistance may also depend on high D2R density (Figure 3). Accordingly, many studies have explored D2R changes after long-term antipsychotic treatment. Early studies showed an increase in striatal D2R binding in rats treated for up to one month with typical antipsychotics, such as haloperidol [106,107,108,109]. However, after long-term antipsychotic treatments, the density of D2Rs in the rat striatum generally increases by 10–40% only. This limited increase appears not to be sufficient to quantitatively explain the behavioral effects of dopamine supersensitivity [110]. Moreover, several reports of dopamine supersensitivity in rats without any significant change in D2R density have been published [111,112]. Notably, it has been recently observed on cellular lines that multiple antipsychotics, but not clozapine, cause time and concentration-dependent increase in surface D2R expression (Table 4) [113]. The antipsychotic-mediated enhancement of D2R cell surface expression depends on antipsychotic binding to an intracellular D2R pool and enhancement of its translocation and surface insertion [113]. This mechanism may explain antipsychotic-induced dopamine supersensitivity and clozapine superiority in resistant phenotype. Nonetheless, a replication of these findings in other paradigms is necessary.

Taken together, these reports indicate that the increase in striatal D2R density may not fully explain the neurobiology of acquired dopamine supersensitivity, although it may represent a valuable pathophysiological mechanism to account for when prescribing prolonged antipsychotic treatments.

An innovative model that may account for acquired dopaminergic supersensitivity is the so-called “cooperativity model” [114,115] (Figure 3). This model relies on the observation that D2Rs may aggregate in oligomers (composed of two-to-four D2Rs), in which they are in the high-affinity state unless unoccupied by the agonist. The binding of the agonist to one of the D2Rs, in turn, reduces the affinity for the agonist of the other unoccupied receptors composing the oligomer (i.e., the unoccupied receptors switch to a low-affinity state). This phenomenon has been defined as “negative cooperativity” [89]. Notably, a high proportion of D2^High^ receptors has been observed in the striatum of supersensitive animals, possibly as a consequence of impaired negative cooperativity in D2R oligomers [88]. Accordingly, it has been reported that: i) animal models of dopamine supersensitivity are systematically linked to an elevation of D2^High^ receptor proportion; and ii) prolonged antipsychotic treatments lead to a considerable increase (i.e., two to four-fold) of the proportion of D2^High^ receptors in the striatum [89]. Considering these clinical and preclinical data together, it has been proposed that impaired negative cooperativity may represent a pathophysiological mechanism of acquired resistance to antipsychotic treatment.

However, a recent study has completely reconsidered the putative neurobiology of this condition (Table 4) [116]. Indeed, the authors manipulated animals to obtain a model of long-term antipsychotic-induced D2R blockade and behavioral sensitization. In this model, they did not find any increase in D2R levels or sensitivity within the ventral striatum [116]. On the contrary, the major neuropathological lesion that authors observed was hyperexcitability in the ventral striatum subpopulation of D2R-expressing medium spiny neurons (MSN), which, in turn, was mainly driven by the insertion of Ca^2+^-permeable α-amino-3-hydroxy-5-methyl-4-isoxazolepropionic acid receptor (AMPAR) and loss of D2R-dependent inhibitory postsynaptic currents [116]. According to these findings, long-lasting synaptic plasticity rearrangements leading to an increased glutamatergic transmission onto D2-MSNs, but not D1-MSNs, may be the more relevant neurobiological mechanism to cause dopamine supersensitivity and therefore acquired resistance to antipsychotics. If supported by additional studies, this result may pave the way to multiple unprecedented therapeutic strategies to prevent or overcome dopamine sensitization and loss of antipsychotic efficacy during the course of the treatment.

**Table 4:** Comparison of treatment resistant and treatment responder patients/preclinical paradigms evaluating D2R-related biological mechanisms of non-response to antipsychotics. AESOP-10: Aetiology and Ethnicity in Schizophrenia and Other Psychoses.

Study Design	Model/Subjects	Methodology	Main Outcomes	References
Longitudinal population study	323 FEP patients (at baseline and after 10-year follow-up)	All patients were drug-naïve or recently treated at baseline and medicated at the endpoint Patients belonged to the AESOP-10 cohort. Medication and clinical history were assessed longitudinally TRS definition based on NICE 2014 criteria (Clinical guideline 178)	Most treatment resistant patients do not respond to antipsychotic treatment, even at the time of FEP It is not clear whether FEP may be already affected by dopamine supersensitivity	[97]
Retrospective population study	246 FEP patients (with a follow-up period of 5 years)	All patients were drug-naïve or recently treated at baseline and medicated at the endpoint EPCRs database interrogation allowed to reconstruct retrospectively medication and clinical history TRS definition based on: 1. clozapine use during the course of the illness; or 2. NICE 2014 criteria (Clinical guideline 178)		[96]
Cross-sectional study	611 patients with schizophrenia or schizoaffective disorder (DSM-IV-TR) (147 TRS of which: 106 DSP 41 without DSP)	In outpatient and inpatient settings, patients suffering from chronic schizophrenia and in active antipsychotic treatment TRS diagnosis was defined according to the Broadest Eligibility Criteria [117] DSP diagnosed according to research criteria proposed by Chouinard [99]		[98]
Retrospective population study	265 patients with schizophrenia or schizoaffective disorder (DSM-IV-TR) (treatment resistant and treatment responders)	In outpatient and inpatient settings, patients suffering from chronic schizophrenia and in active antipsychotic treatment TRS diagnosis was defined according to the Broadest Eligibility Criteria [117] DSP diagnosed according to research criteria proposed by Chouinard [99]	DSP has been regarded as a pivotal factor in treatment resistant schizophrenia, at least the acquired subtype	[101]
In vitro preclinical study	Cultures of prolactin-secreting pituitary-derived MMQ, and HEK293T cells	ELISA and Western blot analysis	Multiple antipsychotics, but not clozapine, cause time and concentration-dependent increase of surface D2R expression.	[113]
In vitro preclinical study	HEK293T Cells	NanoBiT^®^, and Western blot analysis	Distinct D2R antagonists may differently affect D2R dimerization levels, which may have effects on downstream postreceptor signaling and may putatively contribute to explain differences in response to antipsychotics.	[118]
Preclinical study	WT rats and transgenic mice	In vivo Ca^2+^ imaging, Western blot analysis, ex vivo electrophysiology	Behavioral supersensitivity results from mechanisms of synaptic plasticity, insertion of Ca^2+^-permeable AMPA receptors, and loss of D2R-dependent IPSCs in the NA. The chemogenetic restoration of IPSCs in D2-MSNs has been shown to prevent supersensitivity	[116]

TRS = treatment resistant schizophrenia; FEP = first-episode psychosis; EPCRs = electronic psychiatric clinical records; DSP = dopamine supersensitivity psychosis; WT = wild type; AMPA = α-amino-3-hydroxy-5-methyl-4-isoxazolepropionic acid; IPSCs = induced pluripotent stem cells; NICE: The National Institute for Health and Care Excellence; NA = nucleus accumbens; MSNs = medium spiny neurons; ELISA = enzyme-linked immunosorbent assay. SCAN = Schedules for Clinical Assessment in Neuropsychiatry; ICD-10 = Schedules for Clinical Assessment in Neuropsychiatry; DSM-IV-TR = Diagnostic and Statistical Manual of Mental Disorders, 4th Edition, Text Revision.

#### 3.2.4. D2R Dimerization

D2Rs have been described to exist as both monomers and dimers in brain tissues [119]. In human postmortem striatal sections, the expression levels of D2R dimers were significantly increased in schizophrenia patients compared to controls and mood disorder patients [120], while the levels of D2R monomers were significantly decreased. Haloperidol treatment in rats failed to elicit an increase in D2R dimer levels, thereby suggesting that the increases found in schizophrenia striatum were not a consequence of antipsychotic treatment [120]. Likewise, D2R dimer levels were increased and monomer levels decreased in the striatum of amphetamine-induced sensitized state (AISS) rats [120], a model of striatal hyperdopaminergia recalling the purported major dopaminergic lesion in schizophrenia. In these same AISS rats, the proportion of D2R in the high-affinity state was significantly higher than in non-sensitized rats [120], raising the possibility that D2R dimerization may be associated with shifts in the D2R high-affinity state. Theoretically, an abnormal elevation of D2R dimer-containing D2^High^, as a consequence of impaired negative cooperativity, may prevent antipsychotics to revert striatal hyperdopaminergia and cause a non-response. However, there is no evidence to date that treatment resistant patients have a larger proportion of D2R dimers than responder patients.

Intriguingly, it has been recently reported that distinct D2R antagonists may differently affect D2R dimerization levels, which may have profound effects on downstream postreceptor signaling and may putatively contribute to explaining differences in response to antipsychotic agents (Table 4) [118].

#### 3.2.5. D2R-Containing Heteromeric Complexes

**Figure 4:**
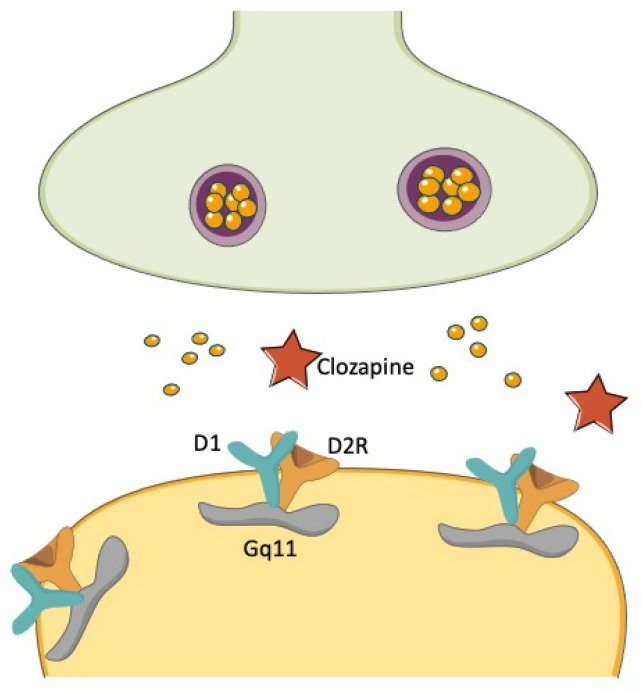
D1R/D2R heterodimerization and the clozapine conundrum. The heterodimerization of D1Rs and D2Rs has been described to yield a powerful stimulation of unique Gq11-mediated signaling, which is distinct from the signaling pathways activated by D1Rs and D2Rs, respectively, when stimulated separately. The effectiveness of clozapine in treatment resistant schizophrenia has been conceptualized to depend on the simultaneous occupancy of D1Rs and D2Rs and the subsequent inhibition of their effects on this unique transduction pathway.

**Figure 5:**
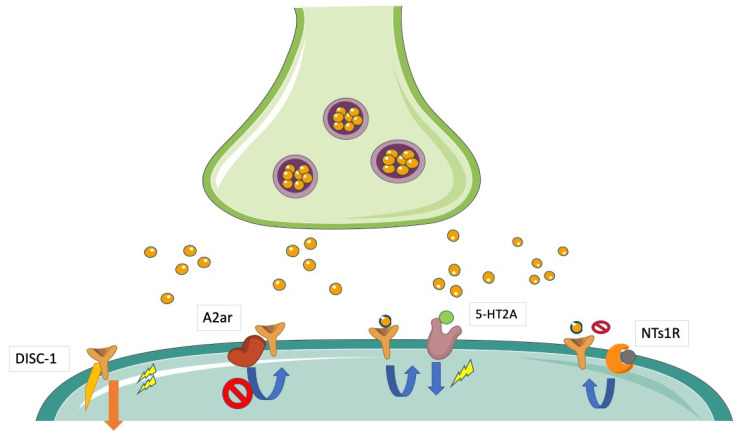
Modulation of D2R-mediated signaling by heterodimerization or intracellular cross-talk. D2Rs may directly or indirectly interact with different transmembrane or intracellular partner proteins. These interactions are regarded to affect levels of downstream signaling and are supposed to modify the response to antipsychotic agents. The interaction with DISC-1 potentiates D2R-mediated signaling, whereas putative interactions with A2AR and NTs1R have been reported to decrease it. Moreover, there is evidence of a cross-talk between D2R and 5-HT2AR, which enhances D2R-mediated downstream signaling.

D2Rs have been supposed to interact, either physically or functionally, with multiple dopaminergic and non-dopaminergic receptor subtypes [121,122]. These interactions have been demonstrated in cellular systems or animal models, while their actual existence in humans has not been confirmed (Figure 4 and Figure 5). Thereby, the relevance of disease pathophysiology and pharmacological action is yet to be determined. Nonetheless, aberrant functions of putative D2R-containing heteroreceptor complexes is an interesting field of research with alleged relevance for treatment resistant conditions.

A functional D1/D2R heteromer complex has been initially reported by co-immunoprecipitation studies from rat and human striatum [123]. Co-activation of both receptors in the context of this complex triggered a unique G_q_-mediated intracellular signaling leading to increased release of intracellular calcium [124]. This unique signaling was distinct from those elicited by the constituent receptors once activated separately [124]. The D1/D2R heteromer complex also showed unique cell surface localization, internalization, and transactivation features [125]. It has been hypothesized that D1/D2R heteromers located in cell bodies and presynaptic terminals attenuate the phosphorylation of the GluR1 AMPARs by modulating Ca^2+^/calmodulin kinase II signaling directly in the *nucleus accumbens* [126]. Of interest, the upregulation of D1/D2R heteromers has been found in the striatum of amphetamine-treated rats and postmortem studies of schizophrenia patients also in the globus pallidus, suggesting its involvement in the psychopathology of schizophrenia and other disorders involving elevated dopamine transmission [126]. D1/D2R heteromer complex signaling was found attenuated by the typical antipsychotic-like raclopride [126]. Moreover, clozapine was found to uncouple D1/D2R heteromer complex in the high-affinity state [127], suggesting that at least clozapine efficacy may be linked to its action on this heterocomplex (Figure 4). However, further work has strongly argued against the actual existence of the D1/D2R heteromer complex in adult rat striatum [128].

One widely studied heterocomplex is formed by adenosine A2A receptors (A2ARs) and D2Rs (Figure 4). The existence of this complex has been demonstrated in cultured living cells [129] and in rat ventral striatum [130]. A2AR activation has been described to reduce agonist binding to striatal D2Rs and attenuate D2R-mediated effects [131]. Therefore, the A2AR function in the context of the A2AR/D2R heteromer may be to dampen D2R signaling [132]. According to this view, it has been postulated that a disruption of this interaction may have causal relevance in the pathophysiology of schizophrenia [121] and may consequently play a major role in antipsychotic action. Recently, a significant reduction of A2AR/D2R heteromers in the caudate nucleus of schizophrenia subjects has been described in a postmortem study [133]. This outcome in humans was confirmed preclinically in the phencyclidine (PCP) model of psychosis, where authors observed an upregulation of D2Rs but a significant reduction of striatal A2AR/D2R heteromers, which was counteracted by chronic haloperidol or clozapine treatment [133]. Moreover, a differential impact of antipsychotics on temporal dynamics of A2AR/D2R heteromer expression in HEK293 cells has been described. Namely, 2-h cell incubation with haloperidol and aripiprazole did not affect heteromer content, while incubation with clozapine diminished its content in a concentration-dependent manner [134]. On the other hand, heteromer levels were significantly increased by 16-h incubation with haloperidol and aripiprazole but not with clozapine [134]. More studies are needed to make inferences on the putative functional implications of these preliminary reports.

In membranes from HEK293 cells transfected with both D2 and 5-HT2A receptors (5-HT2ARs) and in mouse striatum, the D2R agonist quinpirole induced a marked increase in the affinity of the serotonergic agonist 1-[2,5-dimethoxy-4-iodophenyl]-2-aminopropane (DOI) for 5-HT_2A_Rs [135], demonstrating a functional cross-talk between these two receptors (Figure 4). Notably, the increased DOI affinity for 5-HT_2A_Rs by quinpirole was lost in membranes expressing 5-HT_2A_Rs only. The existence of D2/5-HT_2A_R heteromers was demonstrated by co-immunoprecipitation assays on membranes from HEK293 cells expressing both receptors [135]. Remarkably, DOI-induced agonist activity of 5-HT_2A_Rs was enhanced in the presence of D2Rs but reduced when D2Rs were stimulated by their agonist [135], indicating a complex cross-talk rather than mere reciprocal stimulation. Furthermore, the stimulation of 5-HT2AR/D2R heteromers with D2R agonists has been shown to be suppressed by the co-administration of 5-HT2A-agonists, indicating a 5-HT2AR-mediated trans-inhibition of D2Rs [136] and suggesting to explore this heteromer as a potential target for new therapeutic strategies for schizophrenia treatment. The behavioral effects of haloperidol in reverting hyperlocomotion in MK-801-treated mice were also lost in transgenic mice lacking 5-HT_2A_Rs [135].

Moreover, a putative D2R-5HT_1A_R heterodimer has also been described in the mouse frontal cortex [137,138]. Notably, low-dose subchronic clozapine increases the levels of D2R-5HT_1A_R heterodimer in the prefrontal and frontal cortices of the mouse brain, while subchronic haloperidol lowers them [138]. Whether this heterodimer is present in human brains and which functional relevance, also in terms of putative differential neurobiological effects of clozapine compared to conventional antipsychotics, is yet to be determined.

Neurotensin (NT) has been proposed to modulate dopaminergic transmission by a direct antagonist interaction between Neurotensin Receptor 1 (NTS1Rs) and D2Rs (Figure 5). An NT-induced reduction in D2R agonist affinity has been found in both dorsal and ventral rat striatum and may reflect direct allosteric NTS1R/D2R interactions [139].

These elements appear to indicate that the cross-talk between D2Rs and other receptors may be integral to antipsychotic action. Dysfunctions of these cross-talks may be theoretically responsible for the lack of antipsychotic efficacy in treatment resistant patients. Despite the fascinating implications that the research may have for a deeper understanding of schizophrenia molecular pathophysiology and antipsychotic actions, the major flaw is represented by the lack of a clear demonstration of the existence and functional relevance of D2R heteroreceptor complex in humans, although solid evidence of their existence in vivo in rats has been provided [140]. This field indubitably represents one of the most promising focuses of research for the next years.

According to the view that greater effort should be provided to demonstrate the occurrence of D2R-containing heterocomplexes in schizophrenia patients, one study has postulated the existence of a D2R/Disrupted in schizophrenia 1 (DISC1) heterocomplex in humans (Figure 4). *DISC1* is a known susceptibility gene for schizophrenia [141], whose products behave as a scaffolding protein interacting with many signaling molecules, including glycogen synthase kinase-3 (GSK-3) [142]. The D2R/DISC1 complex was found to significantly increase in schizophrenia postmortem striatal tissues compared to controls [143]. The potential confounding role of antipsychotic treatment on D2R/DISC1 levels was ruled out by the observation that acute and chronic haloperidol treatments significantly diminished, rather than increased, D2R/DISC1 interactions in mice [143]. Notably, quinpirole activation of D2Rs significantly increased the D2R/DISC1 interaction in rat striatal neurons, an effect that was blocked by haloperidol [143]. DISC1 was found to facilitate the D2R-mediated reduction of GSK-3 Ser 21/9 phosphorylation by quinpirole and to inhibit agonist-induced D2R internalization [143]. Disruption of the D2R/DISC1 interaction by a specifically designed interfering peptide prevented the D2R-mediated modulation of GSK-3 Ser 21/9 phosphorylation [143]. These data indicate that DISC1 was recruited by agonist stimulation of D2Rs and facilitated or even potentiated downstream signaling initiated by this receptor. Therefore, the D2R/DISC1 heterocomplex may either contribute to or reinforce a condition of hyperdopaminergia in schizophrenia and possibly be responsible for the limited response to antipsychotic treatments. Consistently with these suggestions, the disruption of the D2R/DISC1 interaction has been found to reverse hyperactivity and prepulse Inhibition (PPI) aberrations in multiple rodent models of psychosis [143]. Finally, recent studies have demonstrated the involvement of DISC1 × D2R protein-protein interactions in the mechanisms of cognitive and synaptic plasticity and their modulation as pharmacological targets, contributing further insight into the molecular–cellular mechanisms of antipsychotic drugs [144].

#### 3.2.6. D2Short/D2Long Levels

D2Rs are formed by two molecularly distinct isoforms, i.e., the short (*D2S*) and long (*D2L*) ones, which are generated by alternative splicing of the same gene [145]. It has been reported that the D2S isoform exerts presynaptic D2R-mediated functions [146], while the D2L isoform exerts postsynaptic-mediated effects [145]. This specificity is likely linked to D2L and D2S propensity to interact with diverging G proteins and different downstream signaling pathways [145]. Notably, transgenic mice expressing the short but not the long D2R isoform still preserved their D2R-mediated autoreceptor inhibitory function [147], thus supporting the view that D2S receptors exert inhibitory feedback on presynaptic dopamine release.

In postmortem tissues, mRNA expression of the D2S isoform and the D2S/D2L ratio was significantly increased in the dorsal PFC of schizophrenia patients compared to controls [148]. However, the difference between responder and non-responder patients was not made. No significant differences were found in the caudate-putamen, which has been considered the most relevant brain region for antipsychotic effects on dopamine neurotransmission.

An earlier study showed the differential contribution of D2S vs. D2L isoforms to the actions of antipsychotics [149]. Specifically, the typical antipsychotic raclopride was less potent in inhibiting locomotor activity and eliciting catalepsy in transgenic mice lacking the D2L isoform (*D2L^−/−^*) compared to wild-type mice. On the other hand, the atypical antipsychotic clozapine was equally effective in *D2L^−/−^* and wild-type mice [149]. Consistently with this study, haloperidol has been described to exert some of its biological actions by preferentially targeting D2L receptors [150]. These results may indicate that antipsychotics exert part of their action by targeting more or less selectively one of the two isoforms. However, the putative relevance of these observations for treatment resistance in schizophrenia patients is still elusive.

In humans, an intronic single nucleotide polymorphism in the *DRD2* gene (i.e., *rs1076560*, *G* > *T*) has been observed to shift mRNA splicing to the two functionally distinct isoforms [151]. Specifically, the *T* allele has been associated with a reduced expression of the D2S isoform relative to the D2L in the PFC and striatum of both schizophrenia patients and controls [151]. The *T* allele has also been associated with reduced activity of prefrontal-striatal pathways and impaired working memory performances in schizophrenia patients [152]. In a study using single photon emission computed tomography (SPECT) with [^123I^]IBZM (which binds primarily to postsynaptic D2Rs) and [^123I^]FP-CIT (which is known to bind to presynaptic dopamine transporters, whose activity and density is also regulated by presynaptic D2Rs), a reduced radioligand binding in the caudate-putamen of healthy subjects carrying the *T* allele compared to homozygous *G* allele carriers has been found [153]. *T* allele carriers also had a significant negative correlation between striatal D2R-mediated signaling and activity of the PFC during working memory tasks [153]. One possible explanation of these results is that reduced D2S levels in *T* carriers may increase synaptic dopamine levels due to reduced autoinhibitory control by presynaptic D2Rs. In turn, heightened synaptic dopamine may compete with radioligands for binding to D2Rs [153]. The increased dopaminergic load in the striatum may be responsible for impaired prefrontal-striatal pathways and defective cognitive performances. Indeed, it has been conceptualized that striatal hyperdopaminergia may underlie PFC-dependent cognitive dysfunctions [154]. Accordingly, a seminal work has demonstrated that overexpression of striatal D2Rs causes persistent abnormalities in PFC functioning, including cognitive performances [155].

In agreement with these reports, a recent study has demonstrated that the antipsychotic risperidone may ameliorate executive functions in both schizophrenia patients and mice carrying a genetic variation of the *Dysbindin* gene reducing dysbindin-1 levels [156]. Dysbindin-1 is a synaptic protein implicated in synaptic vesicles and receptor recycling that is known to alter D2R availability [157]. Based on these functions, dysbindin-1 modulates PFC activity and triggers schizophrenia-like behaviors via a D2R-mediated pathway [158,159]. Notably, the D2S/D2L ratio in the dorsolateral PFC was found to increase in schizophrenia patients with reduced dysbindin-1 expression who tested positive in antipsychotic screening [156]. Therefore, the association between low dysbindin-1 levels and antipsychotic treatment led to enhanced presynaptic D2R function within the PFC, which in turn was predicted to improve executive functions in these patients [156]. According to these reports, abnormally high, putatively genetic dysbindin-1 levels may cause diminished presynaptic D2R activity, which may theoretically lead to higher synaptic dopamine levels and poor response to antipsychotics.

These reports are the first steps in the comprehension of the pathophysiology of D2S/D2L isoforms in schizophrenia. They appear to comply with the view that one common dopaminergic dysfunction in schizophrenia may be enhanced presynaptic dopamine release in the striatum. However, the role of these isoforms in antipsychotic actions and putatively in treatment resistance is yet to be established.

#### 3.2.7. Extrastriatal D2Rs

Some lines of research have sought to evaluate whether extrastriatal D2Rs may also play a role in psychosis. Accordingly, several neuroimaging studies have demonstrated that D2R density in multiple extrastriatal sites, including the anterior cingulate cortex, the thalamus, the temporal cortex, and the midbrain, is significantly lower in antipsychotic-free schizophrenia patients compared to controls [160,161,162,163]. Behavioral correlates of these neuroimaging phenotypes are difficult to explicate at the moment. However, there is some evidence that differences in D2R density in extrastriatal sites may be associated with cognitive tasks, such as reward valuation [164] or executive functions [165], as well as with excitement symptoms [166], which may all be part of the complex and heterogeneous clinical phenotype of schizophrenia.

A few studies have evaluated whether the modulation of extrastriatal (mostly cortical) dopamine receptors by antipsychotics may be related to symptom improvement. A SPECT study used the D2/D3R ligand [^123^I]epidepride to evaluate D2/D3R binding potential in antipsychotic-naïve first episode schizophrenia subjects at baseline and after a 3-month treatment by risperidone or zuclopenthixol. No significant association was found between D2/D3R occupancy in extrastriatal sites and improvement in the Positive and Negative Syndrome Scale (PANSS) positive subscale score at the 3-month follow-up evaluation [167]. However, a significant positive correlation between baseline D2/D3R availability in the cortex and post-treatment improvement in positive symptoms was found, although this was limited to the risperidone group only [167]. Notably, in a previous study on the same cohort, no significant differences in frontal D2/D3R availability between patients and controls were found [168], therefore excluding the possibility that D2/D3Rs may be higher in patients as an inherent effect of the disease. In summary, patients with higher D2R levels in the cortex appear to be more responsive to antipsychotic agents, although this was seen with risperidone but not with zuclopenthixol. These findings replicated what was observed in striatal sites [169].

### 3.3. Presynaptic Dopamine Synthesis

#### Baseline and Stimulated Dopamine Levels

**Figure 6:**
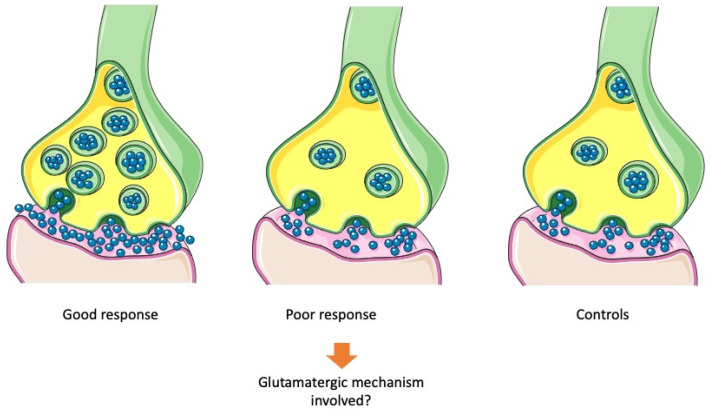
Putative presynaptic molecular mechanisms of treatment resistance. Antipsychotics may bring back abnormally high presynaptic dopamine levels in psychotic patients to physiological levels. Presynaptic hyperdopaminergia is supposed to be a major mechanism of good response to treatment. On the other side, poor response to antipsychotic treatment in treatment resistant patients has been associated with non-significantly different levels of presynaptic dopamine compared to non-affected controls.

Pivotal studies on the amphetamine-mediated displacement of radiolabeled D2/D3R ligands have shown that amphetamine-induced efflux of dopamine in the striatum is abnormally high in schizophrenia patients, even prior to antipsychotic treatment [170,171,172]. [^123I^]IBZM binding to striatal D2Rs was also significantly higher in schizophrenia patients compared to healthy controls after dopamine depletion by alpha-methyl-para-tyrosine [169], indicating higher D2R availability in schizophrenia patients than in controls. Accordingly, schizophrenia patients exhibited a significantly higher increase in D2R availability as a percentage of baseline levels [169]. Changes in D2R availability after dopamine depletion were considered to be indirectly indicative of synaptic dopamine levels at baseline, based on the consideration that the higher the synaptic dopamine levels, the higher the percentage of D2Rs available for radioligand binding after dopamine depletion. Therefore, dopamine appears to occupy a larger proportion of striatal D2Rs in schizophrenia patients than in controls at baseline, and dopamine release by stimulation of presynaptic sites appears to be heightened in schizophrenia compared to controls. These results strongly suggest that presynaptic levels of dopamine in schizophrenia patients’ striatum may be higher than in controls (Figure 6). Indirect support and expansion to this suggestion have recently come from a preclinical study [173]. Intriguingly, the selective activation of dorsal striatum dopamine transmission in transgenic mice impaired working memory and social interaction, which are behavioral processes related to the negative and cognitive symptoms of schizophrenia. These behavioral deficits were not reverted by haloperidol, while they did not occur following treatment with the non-selective brain-wide dopamine releaser amphetamine [173]. These findings suggest that non-responsive cognitive and negative symptoms of schizophrenia may also depend on striatal hyperdopaminergia, putatively restricted to the area of the dorsal striatum, which has large connections with the PFC [173].

According to these reports, studies using radiolabeled 3,4-dihydroxyphenylalanine (DOPA, a powerful marker of presynaptic vesicular dopamine stores) consistently demonstrated an accumulation of presynaptic dopamine in schizophrenia patients’ striatum [174,175]. Notably, the accumulation of presynaptic dopamine was also found in antipsychotic-naïve patients [176,177], thereby implicating that accumulated dopamine may not be a consequence of antipsychotic treatments, whereas it may putatively predispose to antipsychotic response (Figure 5).

In agreement with these suggestions, it has been observed that DOPA levels were significantly higher in both first-episode psychotic and at-risk-mental-state patients compared to controls [68]. It has been demonstrated that dopamine release was also enhanced in both clinical-high-risk and antipsychotic-naïve schizophrenia patients compared to healthy volunteers in a model of psychosocial stress [178]. These studies supported the view that abnormally high presynaptic levels of dopamine are one of the major neurobiological lesions in schizophrenia (Figure 5).

Notably, a meta-analysis of PET and SPECT studies investigating DAT density in schizophrenia patients’ striatum found no significant differences with matched controls, indicating that the density of striatal dopamine terminals does not differ between patients and controls [179] and rejecting the hypothesis that presynaptic hyperdopaminergia may be due to the increased number of dopamine terminals rather than dopamine accumulation in presynaptic sites (Table 5).

In summary, there is consistent evidence that accumulation of presynaptic dopamine may underlie psychotic symptoms and even predate the onset of these, or of those predisposing to relapse.

Under these lines, in an early study, in the decrease in positive symptoms (PANSS positive subscale score changes after 6 weeks), antipsychotics correlated with a larger percentage increase in D2R availability triggered by presynaptic dopamine depletion [169]. This finding indicated that response to antipsychotics may be larger in patients with higher baseline dopamine levels. Therefore, high dopamine levels at baseline may predispose to (or predict) antipsychotic response.

In agreement with this earlier study, a further SPECT study with [^123^I]iodobenzamide showed that there was a significant negative correlation between low striatal D2R binding potential at baseline (which was considered to indicate higher synaptic dopamine levels) and amelioration of positive symptoms after a 6-week treatment with amisulpride in antipsychotic-naïve schizophrenia patients [180]. Responder patients had significantly lower D2R binding potential than non-responder ones, a finding that provided indirect support to the observation (discussed in the next paragraph) that treatment resistant patients had decreased dopamine synthesis capacity compared to responder patients [181]. Moreover, a putative biological signature differentiating treatment resistant and treatment responder patients and involving dopamine synthesis capacity has been recently described in a [18F]-DOPA PET and diffusor tensor imaging (DTI) combined study [182]. Specifically, treatment responder patients exhibited a significant negative correlation between the dorsolateral PFC-associative striatum connectivity and dopamine synthesis capacity of the associative striatum, while no significant correlation was found in treatment resistant patients and healthy controls [182]. In partial agreement, another multimodal study reported that treatment responder patients had a negative correlation between prefrontal grey matter volume and striatal dopamine synthesis capacity, but this was not evident in treatment resistant subjects (Table 5) [183]. While these results support the idea that striatal dopamine disturbances may be driven by cortical abnormalities in schizophrenia, the lack of correlation in treatment resistant patients may be consistent with the suggestion that TRS and non-TRS are two neurobiologically-separated entities [184], at least in terms of dopamine dysfunctions.

An intriguing and somewhat different point of view has been recently suggested [185,186], based on the observation that antipsychotic efficacy in rat models declined in concert with extracellular striatal dopamine levels rather than insufficient D2R occupancy [186]. Indeed, antipsychotic efficacy was associated with a suppression of DAT activity via direct blockade [186], while the loss of efficacy was associated with reduced dopamine neuron firing and restored dopamine transporter activity [186]. Therefore, antipsychotic efficacy may be driven by dynamic interactions between endogenous dopamine and presynaptic D2Rs and should depend on high striatal extracellular dopamine [186], whose interaction with presynaptic D2Rs may cause an autoinhibitory control on dopaminergic neurons. Accordingly, and in countertrend with mainstream opinions, Amato and colleagues propose that the antipsychotic-mediated reduction in dopamine reuptake via direct dopamine transporter blockade allows accumulation of dopamine in the synaptic cleft, which increases efficiency by which phasically discharged dopamine triggers presynaptic autoinhibition [185]. Therefore, a therapeutic antipsychotic response would be obtained by blockade of an adequate proportion of D2R and sufficiently elevated extracellular dopamine levels to trigger autoinhibition [185]. On the other hand, non-response to antipsychotics would develop when extracellular dopamine rather than D2R blockade decreases [186], which may occur in conditions where DAT molecules are reduced or hyposensitive to antipsychotics. Furthermore, dopamine transporter (DAT) blockade has been proposed to restore initial synaptic dopamine levels as a therapeutic option to improve the efficacy of antipsychotics in chronic treatment [186], extending the conventional view of postsynaptic D2R antipsychotic blockade to the presynaptic dopaminergic terminal involvement via inhibition of voltage-gated sodium channels and indirect stimulation of the D2R autoreceptor reserve [185]. These findings could be relevant to elucidate antipsychotic-induced synaptic changes and to shed light on DAT blockade as an adjuvant treatment in non-response to antipsychotics condition, such as TRS. Despite the fact that initial experimental support has already been given to this hypothesis [186], stronger evidence should be provided.

Some studies have investigated the effects of antipsychotics on dopamine synthesis capacity in schizophrenia patients based on the consideration that the antipsychotic effect may not be entirely attributed to the blockade of postsynaptic D2Rs. Indeed, similar occupancy of D2Rs in the striatum has been observed in both responder and non-responder patients in an early study [187], suggesting that blockade of striatal D2Rs may be a necessary but not sufficient mechanism of antipsychotic action. Therefore, it may be hypothesized that an antipsychotic-induced reduction of dopamine synthesis capacity may play a role in antipsychotic action (Figure 6).

According to this idea, in a PET study on nine schizophrenia patients, 5-week haloperidol therapy caused a significant decrease in DOPA decarboxylase relative activity in the caudate, the putamen, the thalamus, and the orbital and frontal cortices [188]. These data are in agreement with the hypothesis that chronic antipsychotic regimens may decrease presynaptic dopamine synthesis, in opposition to acute antipsychotic administration, which has been associated with an increase in dopamine synthesis [189]. Therefore, antipsychotic efficacy may be linked to an efficacious reduction of dopamine synthesis capacity.

Data from human studies are reinforced by preclinical observations. Repeated administration of antipsychotics to rodents is known to trigger depolarization block, i.e., a state of dopamine neuron inactivation [190]. In non-manipulated rats, these effects take weeks to occur. On the contrary, in a developmental model of schizophrenia (i.e., prenatal methyl-azoxymethanol acetate exposure) associated with a hyperdopaminergic state, the acute administration of both first and second-generation antipsychotics induced an immediate reduction of the number of spontaneously active dopamine neurons [78]. The activity of dopamine neurons continued to decrease with repeated administrations of both agents [78], thereby mimicking the early and late efficacy of antipsychotics in schizophrenia patients.

As higher dopamine synthesis capacity has been associated with better response to antipsychotics [169], the possibility may arise that non-response to these agents may alternatively depend on exceptionally high striatal dopamine synthesis capacity that cannot be blocked by antipsychotics or on low levels of presynaptic synthesis. Based on these hypotheses, a neuroimaging study sought to evaluate dopamine synthesis capacity by [18F]-DOPA PET scanning in treatment resistant or treatment responder schizophrenia patients as compared to healthy controls [181]. [18F]-DOPA uptake was significantly higher in responder vs. resistant patients in the associative and limbic striatum. Notably, [18F]-DOPA uptake was also higher in responder vs. controls, while no significant differences were found between resistant and controls [181]. The same observations were replicated in a sample of first-episode patients since [18F]-DOPA uptake was significantly higher in responders compared with non-responders and controls and showed significant positive correlations with improvements in PANSS-positive negative and total scores after 4-week antipsychotic treatment [191].

Taken together, these data suggest that non-response to antipsychotics may be due to the fact the psychotic symptoms in these patients are not caused by elevated presynaptic dopamine synthesis (Figure 6). Since patients included in the studies were stable on psychotic symptoms and had not experienced acute symptom relapse in the six months prior to the study, it remains unclear whether the differences in [18F]-DOPA uptake would still be evident during psychotic re-exacerbation. However, it should be noted that treatment resistant patients in this study were extremely more symptomatic than responder patients [181], raising the possibility that the observed differences in dopamine synthesis may depend on symptom levels. To respond to this issue, a later study evaluated dopamine synthesis capacity in treatment resistant vs. responder patients who were also matched for symptom severity [192]. Again, resistant patients showed significantly lower striatal dopamine synthesis capacity than responder ones [192]. These neuroimaging results matched earlier immunocytochemical reports investigating tyrosine hydroxylase (TH) labeling (a marker of dopaminergic synapses) on postmortem tissues from both treatment resistant and treatment responder schizophrenia as well as control brains [193]. Indeed, TH-labeled axodendritic synapses’ density was significantly greater in treatment responders than in either treatment resistant ones or controls [193].

An interesting approach has been to evaluate whether treatment resistant patients had higher heterogeneity in striatal dopamine function compared to treatment resistant patients, a feature investigated in a subgroup meta-analysis of interindividual variance in multiple outcomes of dopamine function [194]. Although TRS and non-TRS were not comparable directly for dopamine synthesis capacity, some indirect inference can still be made. Notably, in the whole group of schizophrenia patients vs. healthy controls comparison, no significant variability was found between groups, but schizophrenia patients had mean lower dopamine synthesis capacity than controls [194]. Higher dopamine synthesis capacity was also found in the subgroup analysis of treatment responsive patients, but not in the subgroup analysis of treatment resistant patients compared to controls, as expected [194]. No significant variability of this measure was found in both treatment resistant and treatment responder groups compared to controls [194].

Subsequent data suggested that treatment resistant patients may have normal striatal dopamine synthesis capacity but elevated anterior cingulate glutamate levels [195], as opposed to responder patients who have elevated striatal dopamine synthesis but normal glutamate levels in the anterior cingulate cortex [196,197]. A recent report indicated that glutamatergic metabolites in the anterior cingulate cortex were also significantly higher in clozapine-resistant TRS patients compared to healthy controls [198]. The possibility of the involvement of non-dopaminergic systems in TRS has also been suggested by a functional resonance imaging study in which treatment responder patients, but not treatment resistant ones, showed a significant attenuation of reward prediction error-related activation, a putative measure of dopamine circuits dysfunction, in multiple brain areas compared to controls [199].

**Table 5:** Comparison of treatment resistant and treatment responder patients/preclinical paradigms evaluating presynaptic dopamine-related biological mechanisms of non-response to antipsychotics.

Study Design	Model/Subjects	Methodology	Main Outcomes	References
Prospective human study	10 schizophrenia patients	[^18^F]N-methylspiroperidol PET	Similar D2R striatal occupancy in both responder and nonresponder patients.	[187]
Cross-sectional human study	18 treatment responders 18 HC	SPECT	Higher dopamine synthesis capacity associated with better response to antipsychotics.	[169]
Postmortem study	Human brain tissue	Immunocytochemical	TH labeled axodendritic synapses’ density was greater in treatment responders than in either treatment resistant ones or HC.	[193]
Cross-sectional human study	12 treatment resistant 12 treatment responders 12 HC	[^18^F]-DOPA PET	Responder patients had lower D2R binding potential than non-responder ones.	[181]
Prospective human study	28 antipsychotic-naïve schizophrenia patients 26 HC	SPECT with [^123^I]iodobenzamide	Negative correlation between low striatal D2R binding potential at baseline and amelioration of positive symptoms after a 6-week treatment with amisulpride in antipsychotic-naïve schizophrenia patients.	[180]
Cross-sectional human study	21 treatment resistant 20 treatment responders	H-MRS	Treatment responders have elevated striatal dopamine synthesis but normal glutamate levels in the anterior cingulate cortex.	[197]
Cross-sectional human study	12 treatment resistant 12 treatment responders 12 HC	[^18^F]-DOPA PET	Treatment resistant showed lower striatal dopamine synthesis capacity than treatment responder ones.	[192]
Cross-sectional human study	21 treatment resistant 21 treatment responders 24 HC	fMRI	Attenuation of reward prediction error-related activation in multiple brain areas of treatment resistant patients compared to HC.	[199]
Cross-sectional human study	27 treatment resistant 26 UTRS 21 treatment responders 26 HC	H-MRS	Glutamatergic metabolites in the anterior cingulate cortex are higher in treatment resistant patients compared to HC.	[198]
Prospective human study	20 FEP or antipsychotic-naïve psychotic patients	[^18^F]-DOPA PET	[^18^F]-DOPA uptake is higher in responders compared to non-responders and HC Significant positive correlations with improvements in PANSS-positive, negative, and total scores after 4-week of antipsychotics.	[191]
Meta-analysis	983 schizophrenia patients 968 HC	Meta-analysis of variance	Higher dopamine synthesis capacity is found in treatment responders, but not in treatment resistant patients compared to HC.	[194]
Cross-sectional human study	40 patients with psychosis	[^18^F]-DOPA PET MRI	Treatment responders have a negative correlation between prefrontal grey matter volume and striatal dopamine synthesis capacity, but this is not evident in treatment resistant patients.	[183]
Multicenter cross-sectional study	92 patients across 4 sites (44 treatment resistant 48 treatment responders) 54 patients at 2 sites (29 treatment resistant 25 treatment responders)	H-MRS [^18^F]-DOPA PET	Treatment resistant patients may have normal striatal dopamine synthesis capacity but elevated anterior cingulate glutamate levels.	[195]
Cross-sectional human study	24 schizophrenia patients 12 HC	[^18^F]-DOPA PET DTI	Dopamine synthesis capacity may represent a putative biological signature to differentiate treatment resistant from treatment responders patients.	[182]

HC = healthy controls; SPECT = single-photon emission computed tomography; TRS = treatment resistant schizophrenia; DOPA = L-3,4-dihydroxyphenylalanine; PET = positron emission tomography; DTI = diffusion tensor imaging; MRI = magnetic resonance imaging; TH = tyrosine hydroxylase; H-MRS = in vivo proton magnetic resonance spectroscopy; fMRI = functional magnetic resonance imaging.

Indirect support for these findings comes from a recent study on gene expression and protein levels of dopamine-related molecules in the postmortem midbrain of schizophrenia patients. In this study, DAT mRNA expression was found significantly decreased in schizophrenia patients compared to controls, in agreement with the hypothesis of a presynaptic dysregulation leading to striatal hyperdopaminergia in schizophrenia [200]. However, DAT protein levels were found to significantly increase in putatively treatment resistant patients (i.e., patients treated with clozapine) compared to patients treated with other antipsychotics [200], which may represent a compensatory adaptation to the blunted dopamine synthesis capacity found in treatment resistant patients.

In partial agreement with these data, in a small sample of schizophrenia patients with comorbid mixed substance dependence, a blunting rather than an increase in presynaptic striatal dopamine release has been observed [201]. These patients, however, still exhibited the patterns of amphetamine-mediated enhanced dopamine release and positive symptoms worsening observed in other samples of schizophrenia patients without comorbid substance dependence [201]. As blunted dopamine release may derive from neurobiological adaptations to prolonged substance use, authors suggest that in these patients, the predominant dopaminergic alteration may not be excessive presynaptic dopamine synthesis but hypersensitive postsynaptic D2Rs to dopaminergic stimulations, possibly related to postreceptor factors implicated in signaling cascade [201].

### 3.4. Postsynaptic D2Rs

Human studies on dopamine-related postsynaptic mechanisms of response to antipsychotics are currently limited by methodological challenges. Preclinical findings have pointed to several potential molecular processes that affect striatal dopamine neurotransmission, and that may be implicated both in the mechanism of action of antipsychotic agents and in the failure of their efficacy, including disrupted mammalian target of rapamycin (mTOR) Complex 2 (mTORC2) signaling [202]; abnormal neuregulin1/ErbB signaling [203]; defective trace amine-associated receptor 1 (TAAR1) activity [204]. However, studies in humans are lacking or limited at the moment. In the following paragraphs, we will focus on the most studied receptor and postreceptor mechanisms implicated in dopaminergic signaling and proposed as molecular targets of antipsychotic action.

#### 3.4.1. Modulation of D2R Internalization

**Figure 7:**
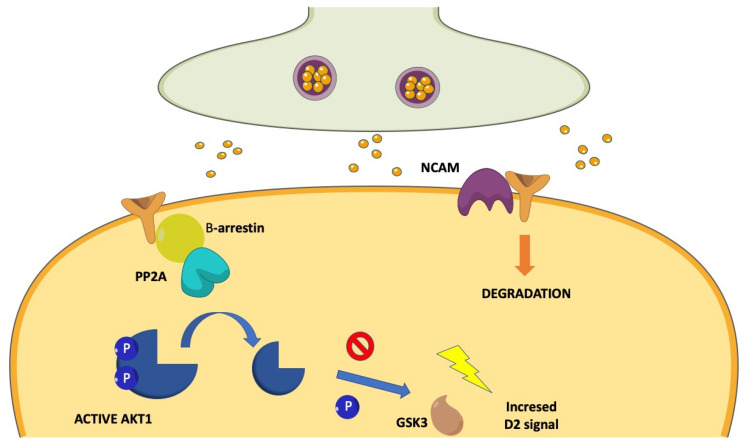
Putative postsynaptic molecular mechanisms of treatment resistance: the β-arrestin-2 pathway and the NCAM. β-arrestin-2 pathway may promote D2R-mediate postsynaptic signaling. The interaction of D2R with β-arrestin promotes the recruitment of PP2A, which in turn disables AKT1. The lack of inactivation of GSK-3 causes hyperactivity of D2R-mediated signaling. Excessive activation of this pathway has been considered a putative mechanism of non-response to antipsychotics. NCAM is a transmembrane protein that interacts with D2Rs and mediates their degradation. NCAM^−/−^ mutant mice showed an increased membrane expression of D2Rs and excessive D2R-mediated postsynaptic signaling, which may counteract the effects of antipsychotic agents predisposing to non-response.

D2R signaling strength may be modulated by internalization and degradation processes. A deficit in internalization/degradation processes may predispose to aberrantly high D2R-mediated neurotransmission, and theoretically, to the lack of response to antipsychotic agents (Figure 7).

The neural cell adhesion molecule (NCAM) is a transmembrane postsynaptic protein that interacts with D2Rs via its third intracellular loop [205]. Genetic manipulations that affect NCAM functions have been found to cause schizophrenia-reminiscent behaviors (i.e., impaired prepulse inhibition of startle; enhanced basal locomotor activity; enhanced responses to amphetamine) [206,207]. Moreover, reduced polysialylated NCAM levels and increased NCAM fragments have been found in the brain and cerebrospinal fluid (CSF) of schizophrenia patients compared to controls [208,209]. Notably, NCAM interaction with D2Rs is enhanced on dopamine stimulation and is followed by an NCAM-mediated internalization and degradation of D2Rs [205]. Deficits of NCAM expression in *NCAM^−/−^* mutant mice have been associated with increased membrane expression of D2Rs, excessive D2R-mediated postsynaptic signaling, enhanced locomotor activity in response to dopamine-agonists, and lower response to a D2R antagonist in terms of locomotor activity reduction [205]. Therefore, it can be speculated that the production of defective NCAM molecules may be one possible postsynaptic mechanism predisposing to antipsychotic resistance (Figure 7).

Notably, the *rs1801028* polymorphism of the *DRD2* gene corresponds to a *Ser/Cys* alternative phenotype in the aminoacidic sequence located in the area of NCAM-D2R interaction. This polymorphism has been considered a risk factor for schizophrenia, although recent meta-analysis did not confirm this view [210] and has been investigated in relation to response to risperidone [211]. Compared to wild-type mice carrying a Ser residue in position 311 of the aminoacidic sequence, *311Cys* mutants showed a 35% decrease of D2R binding to NCAM [205], which may correspond to an increase in surface D2Rs and possibly to augmented D2R-mediated responses to dopamine. According to this possibility, it has been observed that *Cys* allele carriers had significantly higher PANSS total and subscale scores compared to *Ser/Ser* patients [212]. Since patients included in this study were all under antipsychotic treatment, the differences in PANSS scores may also depend on inefficacious treatment.

#### 3.4.2. β-arrestin Signaling

Striatal D2Rs have been considered to exert part of their downstream action in a cyclic adenosine monophosphate (cAMP)-independent manner by the formation of a signaling complex comprising AKT1, protein phosphatase 2 (PP2A), and β-arrestin-2 [213]. Formation of this complex in response to dopamine agonists requires β-arrestin-2, which in this case appears to promote D2R-mediate postsynaptic signaling, rather than contributing to terminating it [214]. D2R-mediated recruitment of β-arrestin-2 and PP2A leads to the inactivation of AKT1 by dephosphorylation of distinct target sites [214], which in turn prevents the inactivation of the constitutively active GSK-3 by AKT1-mediated phosphorylation and contributes to the expression of dopamine-related behaviors [213]. Therefore, AKT1 hypoactivity and GSK-3 hyperactivity may represent postsynaptic mechanisms of heightened D2R-mediated dopaminergic tone, as is supposed to occur in schizophrenia (Figure 7). Disturbances in the function of these molecules may also represent postsynaptic mechanisms of non-response to antipsychotic agents (Figure 7). Notably, significant alterations in gene expression of β-arrestin-1 and 2, along with D2R, metabotropic glutamate receptors (mGluR) 1, and mGluR5, were reported in a G-protein coupled receptor (GPCR) signaling pathway finder study used to assess the action of the TRS gold standard medication clozapine in a model of acute and subchronic ketamine treatment in rats [215], indicating that this molecular machinery may take part to the neuropharmacological actions of clozapine and putatively be among molecular targets for overcoming the resistance to antipsychotic treatment.

Indeed, AKT1 protein levels were found to be significantly reduced in lymphocyte-derived cell lines and in the frontal cortex and hippocampus of postmortem brains of schizophrenia patients compared to controls [216]. Additionally, the relative phosphorylation at Ser9 of GSK-3 was significantly lower in schizophrenia patients’ tissues (both ex vivo lymphocytes and postmortem frontal cortex) compared to controls [216]. AKT1 protein levels were not found to be significantly different in the frontal cortex of haloperidol-treated mice compared to untreated controls [216], presumably excluding the confounding effect of antipsychotic treatment on AKT1 levels. When assessed on a relatively larger sample, a significant association was found between the diagnosis of schizophrenia and an AKT1 haplotype which was predictive of lower AKT1 protein levels [216]. Consistent with this genetic observation, sensorimotor gating was significantly more disrupted by amphetamine in transgenic mice that did not express the AKT1 gene compared to wild-type littermates [216].

Notably, antipsychotic treatments significantly increased AKT1 and GSK-3 phosphorylation in the rodent brain [216,217], possibly indicating that antipsychotics may revert the putative alterations in these two molecules in schizophrenia patients. Accordingly, enhanced striatal AKT activation has been described in *D2R* knock-out mice [218]. Moreover, GSK-3 activation appears to be also regulated by serotonergic receptors since stimulation of 5-HT_2A_ and 5-HT_1A_Rs increased and decreased GSK-3 activation, respectively [219]. Since most antipsychotics are antagonists at 5-HT_2A_Rs and, to a lesser extent, agonists at 5-HT_1A_Rs, GSK-3 may represent a site of convergence of most antipsychotic actions. Remarkably, antipsychotics appear to exert different actions on these molecules. Indeed, levels of phosphorylated AKT1 in the rat frontal cortex rapidly returned to baseline after an acute haloperidol exposure, while AKT1 remained phosphorylated after an acute clozapine treatment [220].

It has been demonstrated that a series of typical and atypical antipsychotics share the property to potently antagonize β-arrestin-2 recruitment by D2Rs after their stimulation by quinpirole [221]. Interestingly, these same antipsychotics range from inverse agonism to full antagonism at the canonical D2R-mediated G_i/o_ protein signaling [221], suggesting that antipsychotics may share inhibition of the D2R-b-arrestin-2 interaction rather than cAMP inhibition as a common molecular mechanism.

A preclinical study has described the generation of unprecedented β-arrestin-biased D2R ligands [222]. These compounds showed potent antipsychotic-like activity without motor side-effects in amphetamine-treated mice, a model of hyperdopaminergia [222], and were able to revert several psychotic-like behaviors in PCP-treated and in *NR1* subunit knock-down mice [223], and two models of NMDA receptor hypofunction-dependent psychosis. Intriguingly, genetic deletion of the gene coding for β-arrestin-2 transformed these compounds into typical antipsychotics, with high liability to induce catalepsy [222].

All these reports indicate that the β-arrestin-2/AKT1/GSK-3 pathway may represent a major target for existing and future antipsychotics [224]. Indeed, the β-arrestin-2/AKT1/GSK-3 pathway has been found to be affected under conditions of D2R hyperstimulation, as those hypothesized in schizophrenia. Modulation of surface receptors to revert these defects may represent one major mechanism of action of antipsychotic agents. However, dopamine-dependent psychotic-like behavioral phenotypes have also been observed in intact D2R animals that exhibited primary impairments in the β-arrestin-2/AKT1/GSK-3 pathway. Transgenic mice overexpressing GSK-3 showed increased general locomotor activity and increased acoustic startle response [225]. Overexpression in mice striatum of a D2R with preferential binding to β-arrestin-2 was associated with potentiation of amphetamine-induced locomotor response similar to that obtained by overexpression of wild-type D2R and significantly higher than that exhibited by mice overexpressing a D2R preferentially binding G_i/o_ [226]. Putative postreceptor dysfunctions in the β-arrestin-2/AKT1/GSK-3 pathway may be responsible for the lack of antipsychotic efficacy.

#### 3.4.3. D2R-Mediated Action on Scaffolding Proteins

Scaffolding proteins are enriched at the postsynaptic density (PSD) of medium-sized spiny neurons, an ultrastructure located in the proximity of membrane surface and whose main biological function is to integrate receptor-mediated signaling with intracellular effectors and to mediate cross-talk among different transduction systems [227]. Despite the fact that PSD is mainly deputed to the integration of glutamatergic signaling, multiple reports have indicated that scaffolding proteins are also implicated in the cross-talk of dopamine-mediated signaling [228]. Moreover, dopamine-mediated stimuli, as well as antipsychotic agents, have been reported to modulate gene expression and protein levels of multiple scaffolding proteins of the PSD [229,230], leading to changes in synaptic architecture and functional connectivity [231]. Moreover, antipsychotics have been reported to differentially affect dendritic spine density and PSD ultrastructure, putatively through the AKT-GSK-3 beta cascade [232].

Striatal expression of the *Homer1a* inducible transcript has been found selectively induced by D2R antagonism [233], and antipsychotic administration differentially regulated *Homer1* gene expression according to the distinct gene isoform, brain region, antipsychotic type, and administration timing [234,235,236,237]. Notably, *Homer1a* has been used as a marker of glutamatergic activity to provide network maps of brain functions in relation to antipsychotic administration [238].

At least one polymorphism in the *Homer1* sequence, i.e., *rs2290639*, has been significantly associated with psychotic symptoms measured by PANSS and response to four-week antipsychotic treatment in a population of schizophrenia patients [239].

Experimental studies suggest that constitutive Homer proteins may facilitate the cross-talk between dopamine and glutamate receptors signaling pathways, while the inducible isoform *Homer1a* may represent a rapidly-induced tool to transiently impair this molecular cross-talk [240] and to prevent excessive neuronal depolarization [241]. These mechanisms may be crucial in the action of antipsychotics, although a direct confirmation of this hypothesis in humans is still lacking. However, it could be supposed that dysfunctions of PSD scaffolding proteins (e.g., mutant hypo/hyperfunctioning isoforms; relative imbalance between PSD molecules) may cause aberrant dopamine-glutamate cross-talk and prevent antipsychotic efficacy.

## 4. Discussion

### 4.1. Methodological Considerations

To date, only a few studies have provided a direct comparison of responder vs. non-responder schizophrenia patients relative to biological underpinnings of the lack of response to antipsychotics. The great part of studies providing such a comparison is from a neuroimaging perspective. Many others do not provide a direct comparison; however, they report data that may be informative of the mechanisms implied in response/non-response to antipsychotics [169]. Only a few studies have addressed the issue of genetic and molecular differences between responder and non-responder patients, while a substantial part of biological information derives from preclinical reports. However, in this case, a major drawback should be taken into account: an animal model of antipsychotic resistance with face, construct, and/or predictive validity is still lacking.

As a result of all these considerations, it should be concluded that research on the field of neurobiological underpinnings of TRS still has a long way to go. Nonetheless, many studies have investigated clinical differences in treatment resistant vs. treatment responder patients [184,242,243], finding sharp separations between the two conditions in many respects. Accordingly, TRS may be categorically distinct from conventionally considered schizophrenia [184] and may depend on different neurobiological mechanisms [196,242].

**Figure 8:**
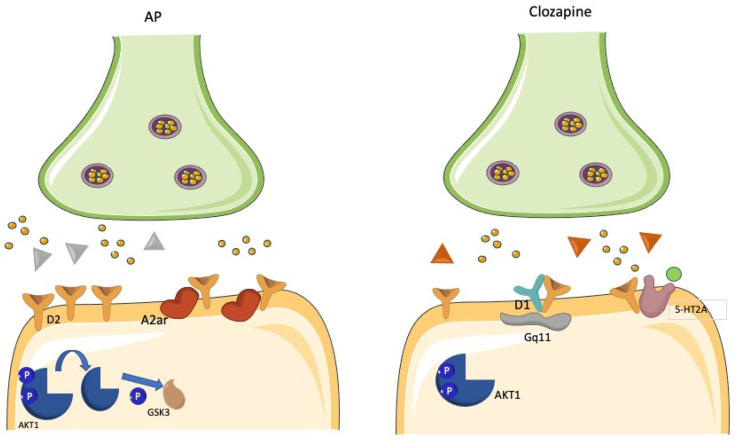
Clozapine is considered the gold-standard intervention in treatment resistant patients, but the unique molecular mechanisms of its superior efficacy in comparison to conventional antipsychotics are still undetermined. The figure depicts some putative mechanisms: D2R/5HT1AR heterodimer levels increase; uncoupling of the D1/D2R heteromer complex in the high-affinity state; lack of time and concentration-dependent increase in surface D2R expression; selective targeting of a unique Gq-mediated second messenger pathway; increased AKT1 and GSK-3 phosphorylation; prevention of the increase in A2AR/D2R heteromer levels, whose formation may contrast the effects of independent A2AR activation in attenuating D2R-mediated signaling.

It has been proposed to re-classify schizophrenia patients according to their response to antipsychotics in three different groups (i.e., responder to conventional antipsychotics; responder to clozapine; non-responder to clozapine) [244]. Such a classification may be extremely useful in clinical settings, however, it is not clear whether biological mechanisms leading to response/non-response in these three sub-groups may be distinct, as the authors suggest [244]. A putative and schematic depiction of clozapine’s unique mechanisms of action in comparison to conventional antipsychotics is given in Figure 8.

Howes and Kapur [245] have proposed a more neurobiologically-oriented model of schizophrenia classification based on dopamine perturbation and treatment response. According to this model, schizophrenia should be differentiated in (at least) two subtypes: type A (i.e., hyperdopaminergic), which is characterized by elevated striatal dopamine synthesis and release capacity and is expected to be responsive to conventional dopamine-blocking agents; and the type B (i.e., normodopaminergic), where these dopaminergic alterations are not present, and that is expected to respond to clozapine but not to conventional antipsychotics [245]. This hypothesis is supported by neuroimaging, biochemical, and postmortem data [193,196,246]. However, none of these studies was specifically designed to test the hypothesis of a differential hyper vs. normodopaminegic state associated with treatment response in schizophrenia patients. Therefore, more focused studies are needed to validate or reject this hypothesis.

Nonetheless, these reports are paving the way for a reconceptualization and a critical appraisal of research strategies used (and to be used) for studying TRS. Recent attempts have been made to unify the nomenclature, to provide operative criteria to define non-response to antipsychotics, and to delineate a distinct TRS syndrome [2]. This attempt is of crucial relevance in order to select homogeneous population samples for conducting more rigorous and reliable studies on the neurobiology of TRS.

### 4.2. Theoretical Considerations

Given the limitations described in the previous paragraph, there is no unitary theory to explain the lack of response to antipsychotics in schizophrenia patients. D2R-related mechanisms have been mostly investigated in preclinical or ex-vivo settings, therefore, the generalizability of these findings is challenging. The main theoretical concept is that non-response to antipsychotics may depend on abnormally high D2R density or functioning. However, to date, there is no direct evidence to confirm this hypothesis. It should also be remarked that no reports have compared responder and non-responder patients, while studies investigating the molecular events linked to response to antipsychotics were carried out in animal models of the disease. Despite early claims on the increased density of D2Rs in the brains of schizophrenia patients [83], subsequent studies and meta-analyses argue against this possibility [11]. One possible alternative explanation is that only a subset of D2Rs may be upregulated in schizophrenia and possibly in TRS conditions. There is convincing evidence that a wide range of preclinical manipulations modeling psychotic phenotypes may cause an upregulation of D2R in the high-affinity state [89]. However, PET human studies have failed to demonstrate a significant difference between patients and controls in D2^High^ density [56,93,95], possibly because of technical drawbacks, since a reliable D2^High^ radioligand has not been manufactured yet. Remarkably, the difference in density of either the whole pool of D2Rs or the subset of D2High has not been investigated in schizophrenia responders vs. non-responders. Indeed, the compensatory increase in postsynaptic D2R levels and/or the enhanced shift of D2Rs to high-affinity states by long-term antipsychotic treatments are regarded as the main pathophysiological mechanisms of the acquired TRS-subtype known as antipsychotic-induced supersensitivity psychosis [100,101]. Again, an increased density of D2Rs and an elevation of D2^High^ receptor proportion (as well as the possibly jointed phenomenon of increased D2R dimer density) have been demonstrated in the striatum of animal models of dopamine supersensitivity [88], whereas reports are lacking in humans.

Treatment resistance may be due to aberrant density/functioning of D2R-containing heterocomplexes, which may preclude antipsychotic interaction with the receptor or may activate unique second messenger cascades that are not functionally targeted by these agents. In recent years, the possibility that D2Rs may form heteromeric complexes with D1Rs has received great attention [247]. D1/D2R heterocomplexes have been found to trigger a unique G_q_-mediated second messenger pathway, which has been reported to be selectively targeted by clozapine [124], thereby providing a molecular explanation for the well-known efficacy of clozapine in non-responder patients. However, the actual existence of this heterocomplex has been strongly questioned in another study [128]. The actual existence in human brains, the pathophysiological role, and the relevance for an antipsychotic response of other postulated D2R-containing heteromeric complexes (i.e., A2A/D2R; 5-HT2A/D2R; NTS1/D2R) is yet to be determined and represents an intriguing field of research.

However, unlike the above-mentioned molecular complexes, Su and co-workers have demonstrated that the DISC1/D2R heterocomplex is enriched in postmortem brains of schizophrenia individuals compared to controls [143], rendering it a compelling candidate among D2R-containing heterocomplexes. Notably, the DISC1/D2R complex forms in response to D2R agonist stimulation and potentiates D2R-mediated downstream signaling via GSK-3 [143], while it is affected by haloperidol treatment. Enrichment of DISC1/D2R complexes may be responsible for a condition of D2R-dependent hyperdopaminergia, which in extreme cases may predispose to poor response to antipsychotics.

Another interesting field of research is represented by putative modulation of antipsychotic efficacy by the D2^Short^ and D2^Long^ variants since there is some evidence that different antipsychotics may differentially target these isoforms [149,150], possibly explaining clinical variations in response among patients. Despite there is no evidence at the moment on the role of D2^Short/Long^ isoforms in regulating the response to antipsychotics in humans, future studies should be aimed at evaluating possible pathophysiological mechanisms of antipsychotic resistance involving these isoforms, including (1) increased levels or biological actions of D2^Short^ receptors, which may be in agreement with observations of Demjaha et al. [196] on lower synaptic dopamine levels in non-responders compared to responders; (2) increased levels or functions of D2^Long^ receptors, which may be consistent with the hypothesis that non-response may depend on postsynaptic mechanisms and may in part comply with observations on increased high-affinity D2R states in schizophrenia and possibly in antipsychotic non-responder; or (3) an imbalance between these two receptor isoforms.

Research on putative postsynaptic mechanisms of non-response to antipsychotics is still in its early steps. Defective D2R internalization, aberrant D2R-mediated downstream signaling, and altered postsynaptic scaffolding protein-mediated glutamate-dopamine interactions are all putative mechanisms that have been proposed to affect D2R-mediated signaling, and that could theoretically predispose to the lack of response to antipsychotic agents. However, most of the evidence is in preclinical settings. However, direct evaluation of treatment resistant patients or comparison between responder and non-responder patients is still lacking.

As a summary of findings, there are multiple intriguing theoretical mechanisms that may explain antipsychotic resistance as an effect of striatal D2R-dependent molecular events. However, to date, no studies have investigated these mechanisms in treatment resistant patients. Additionally, very few studies have been conducted on extrastriatal D2Rs and their relationship with antipsychotic efficacy. Dysfunctional D2R-dependent molecular events may play a role in hyperdopaminergic-based forms of antipsychotic resistance.

Antipsychotic efficacy may be linked to an efficacious reduction of dopamine synthesis capacity. Indeed, clinical studies have demonstrated that prolonged antipsychotic treatment reduces the activity of the DOPA decarboxylase enzyme [188], which is predicted to transform levodopa in dopamine [248]. Preclinical studies show that the inactivation of dopamine neurons is larger in animal models of hyperdopaminergia [78], thereby mimicking the most widely accepted pathophysiological mechanism of psychosis. According to these data, antipsychotics may be more effective in patients whose psychotic symptoms derive from aberrant high dopamine synthesis capacity, which may be successfully impacted by these agents. Therefore, it may be hypothesized that patients whose psychotic symptoms do not derive from high dopamine synthesis capacity may not respond to antipsychotics.

According to these suggestions, it has been observed that DOPA uptake levels in treatment resistant patients were lower than in responder patients and similar to non-affected subjects [181,192]. It has been reported that lower binding potential of a striatal D2R selective radiotracer (i.e., higher baseline synaptic dopamine levels) was correlated to better response to antipsychotic treatment [180] and that baseline binding potential was significantly lower in responder patients than in non-responders [180]. Dopaminergic synapse density also appeared greater in postmortem tissues of responder vs. non-responder schizophrenia patients [193].

These elements support the proposed hyper vs. normodopaminergic schizophrenia dichotomy [245], with the normodopaminergic subtype expected to derive from other, possibly glutamatergic, mechanisms and to be responsive to clozapine. According to Howes and Kapur [245], the most conservative and intuitive explanation should be that antipsychotic agents do not work in some schizophrenia patients simply because their psychosis pathophysiology is not linked to a substantial dopaminergic perturbation. Since antipsychotic agents are all D2R-blocking agents (with the possible but relevant exception of clozapine), they cannot be expected to ameliorate psychotic symptoms that do not depend on dopamine dysfunctions.

However, it is not clear whether all conditions of TRS may be inscribed within the so-called type B schizophrenia. Some conditions may depend on an extremely high hyperdopaminergic state, possibly due to aberrant D2R-mediated and/or post-D2R-mediated mechanisms (see infra). Moreover, as a substantial part of treatment resistant patients is not responsive to clozapine also, it has been proposed that clozapine-resistant patients may be afflicted by a disease with even more different neurobiology than clozapine responder TRS [244]. Namely, it has been demonstrated that minor physical abnormalities, which are indicative of neurodevelopmental failures, are significantly more frequent in treatment resistant than in treatment responder patients [249]. Our recent reports also showed that neurological soft signs (NSS) are more pronounced in treatment resistant patients compared to treatment responders [242], representing one of the most relevant clinical signs to discriminate patients who have developed TRS from those who have not [250]. NSS are minor neurological abnormalities of neurodevelopmental origin that are regarded to depend on aberrant network connections among brain areas [251]. Notably, it has been reported that treatment resistant patients exhibit reduced connectivity between the ventral striatum and substantia nigra and more pervasive disturbance to corticostriatal connectivity compared to matched non-resistant patients [252]. Taken together, these data let us hypothesize that the neurobiological foundations of TRS may be more heterogeneous than only assuming a normodopaminergic state in these patients, putatively implicating further clinical phenotype subdivisions [253,254].

### 4.3. Future Perspectives

Research on the neurobiology of TRS should start from three key points:

(1) better definition of TRS.

Although some efforts have been made in this direction, there is still much to do. While the distinction between responders to conventional antipsychotics, responders to clozapine, and non-responders to clozapine may have large clinical utility [244], some challenges still remain:

(a) How should patients afflicted by dopamine supersensitivity-based TRS be classified? Put another way: may it be useful to parse out acquired TRS from conditions in which antipsychotic resistance occurs since the first episode of psychosis [96,97]?
(b) Should patients with non-responsive non-positive symptoms be included within TRS? Since antipsychotics are not considered to have an efficacious impact on negative and/or cognitive symptoms [255], patients with prominent and non-responsive non-positive symptoms should not be included among TRS or at least should be classified otherwise. Nonetheless, a recent report from our group has demonstrated that disorganization symptoms are as relevant as positive symptoms to categorize schizophrenia patients as TRS with the current operative criteria [256]. It could be hypothesized that treatment resistant patients with prominent non-positive symptoms may fall within the so-called normodopaminergic B subtype of schizophrenia. However, there is no clear evidence that positive symptoms may derive only from hyperdopaminergic state. On the contrary, clozapine, which is not a potent dopamine blocker, is efficacious against positive symptoms in individuals who did not respond to conventional antipsychotics, possibly implicating that their positive symptoms were not dopaminergic in origin. Moreover, suppose patients with non-positive non-responsive symptoms are to be excluded from the TRS definition. In that case, the operative measures to assess non-response should be changed since at the moment the most widely accepted measure of non-response is the lack of substantial reduction of PANSS total score, therefore also including non-positive symptom-related items.
(c) Should non-response to antipsychotics be considered a trans-diagnostic condition rather than limited to schizophrenia? In fact, antipsychotic agents are not intended to treat schizophrenia as a whole but only to ameliorate positive symptoms (or more conservatively to ameliorate those symptoms that derive from high D2R-mediated transmission). Excluding affective psychoses, whose treatment also includes non-antipsychotic agents, the field of non-affective psychoses exhibiting positive symptoms is not merely restricted to schizophrenia. Therefore, the definition of TRS may be excessively narrow and other definitions and inclusion criteria should be provided, e.g., “treatment resistant positive symptom syndrome” which includes patients suffering from non-affective psychosis whose positive symptoms are not ameliorated by antipsychotics.

(2) Carefully designed trials should be carried out to directly compare treatment resistant and treatment responder patients on multiple neurobiological measures, including differential genetic background, mRNA expression patterns, signaling pathways, proteomic and metabolomic events, and functional brain networks. Indeed, there are growing arguments that responder and non-responder patients may suffer from distinct neurobiological lesions [242] and differential neuroimaging correlates of a disrupted functional brain network [67].

(3) Animal models of antipsychotic resistance with at least face and construct validity should be developed, in order to allow more focused molecular research on the underpinnings of treatment resistance.

## Figures and Tables

**Table 1:** Receptors’ affinity profile of antipsychotic drugs.

Antipsychotic	D1	D2	D3	D4	5-HT_1A_	5-HT_1D_	5-HT_2A_	5-HT_2B_	5-HT_2c_	5-HT_6_	5-HT_7_	AchR	α_1_	α_2_	References
Amisulpride	-	++++	++	-	n.t.	n.t.	-	++	-	n.t.	-	-	-	-	[21,22]
Aripiprazole	-	++++	++	+	++	+	+++	++++	+	+	++	n.t.	+	+	[23,24,25,26]
Brexpirazole	+	++++	+++	++	++++	n.t.	++++	+++	++++	+	++	n.t.	++	+++	[24,27,28]
Cariprazine	-	++++	++++	-	+++	n.t.	++	++++	-	n.t.	n.t.	n.t.	+++	+++	[24,27,29]
Chlorpromazine	++	+++	+++	++	n.t.	n.t.	++	n.t.	++	++	++	+	+++	-	[21,30,31]
Clozapine	+	+	+	++	-	-	+++	+++	++	++	++	+++	+++	+	[21,31,32,33,34]
Haloperidol	+	++++	+++	++	-	-	+	n.t.	-	-	-	-	+++	-	[31,32,34]
Lumateperone	++	++	n.t.	n.t.	n.t.	n.t.	++++	n.t.	n.t.	n.t.	n.t.	n.t.	++	n.t.	[35]
Lurasidone	-	+++	++	++++	+++	n.t.	+++	n.t.	-	n.t.	+++	n.t.	+	++	[36,37,38,39]
Olanzapine	++	++	+	++	-	-	+++	++	++	++	-	+++	++	+	[31,32,34]
Paliperidone	++	+++	+++	+	+	-	+++		++	n.t.	+++	n.t.	+++	++	[30,40]
Quetiapine	-	+	-	-	-	-	++	++	-	-	-	-	+++	-	[31,33,41]
Risperidone	+	+++	++	-	-	+	++++	++	++	-	+++	-	+++	++	[32,34,42,43]
Ziprasidone	+	+++	++	++	+++	+++	++++	++	++++	+	++	-	++	-	[44,45]

Abbreviations: 5-HT = serotonin; D = dopamine; Ach = acetylcholine; α = adrenergic; n.t. = not tested; - = minimal to none; + = low; ++ = intermediate; +++ = high; ++++ = very high.

**Table 2:** Antipsychotic dissociation constants (nM) at receptors.

Antipsychotic	D1	D2	D3	D4	5-HT_1A_	5-HT_2A_	5-HT_2B_	5-HT_2c_	5-HT_7_	M_1_	α_1A/B_	α_2A_	α_2c_	References
Amisulpride	1.3	1.8 **	3.2 ***	n.t.	n.t.	n.t.	n.t.	n.t.	n.t.	n.t.	n.t.	n.t.	n.t.	[46,47,48]
Aripiprazole	n.t.	1.8 **	0.8 ^†^	514	1.7 ^†^	n.t	n.t.	n.t.	n.t.	6800 ^†^	26/35 ^†^	n.t.	38 ^†^	[49,50,51]
Brexpirazole	n.t.	0.3^†^	1.1 ^†^	n.t.	0.12 ^†^	0.47 ^†^	1.9 ^†^	n.t.	3.7 ^†^	>1000 ^†^	3.8/0.17 ^†^	n.t.	0.59 ^†^	[52]
Cariprazine	n.t.	0.49 ^†^	0.09 ^†^	n.t.	2.6 ^†^	18.6 ^†^	0.58 ^†^	n.t.	112 ^†^	n.t.	n.t.	n.t.	n.t.	[53]
Chlorpromazine	16.5 *	1.2 **^/$^	1.4 ***	9.6 ^#^	n.t.	2 ^§^	n.t.	n.t.	n.t.	378 ^$^	14.0 ^£^	n.t.	n.t.	[34,49,54,55]
Clozapine	90 *	76 **	190 ***	22 ^#^	123 ± 5	4 ^§^	n.t.	n.t.	42.2 ± 12.0	n.t.	17.5 ± 5.0	147 ± 14	15.6 ± 2.0	[49,55,56,57]
Haloperidol	55 *	0.74 **	8.8 ***	2 ^#^	>1000 (IC_50_ value)	74 ^§^	n.t.	n.t.	>1000 (IC_50_ value)	n.t.	17.9 ± 1.5	>1000 (IC_50_ value)	>1000 (IC_50_ value)	[49,55,57,58]
Lumateperone	52	32	n.t.	n.t.	n.t.	0.54	n.t.	173	n.t.	n.t.	73	n.t.	n.t.	[35,59]
Lurasidone	n.t.	1^†^	15.7	29.7	6.4 ^†^	0.5 ^†^	n.t.	415	0.5 ^†^	>1000 (IC_50_ value) ^†^	47.9 ± 7.8	40.7 ± 7.7	10.8 ± 0.64	[57]
Olanzapine	9.2 *	7.4 **	14 ***	15 ^#^	>1000 (IC_50_ value)	3.4^§^	n.t.	n.t.	n.t.	n.t.	22.1 ± 7.7	n.t.	n.t.	[49,55,57,60]
Paliperidone	670	4	7.50	n.t.	380	0.25	n.t.	n.t.	1.3	3570	4.0	17	n.t.	[61]
Quetiapine	290 *	140 **	240 ***	2000 ^#^	1000 ^†^	135 ^§^	n.t.	n.t.	1800 ^†^	1100 ^†^	22/15 ^†^	n.t.	29 ^†^	[49,60,62]
Risperidone	42 *	1.09 **	3.5 ***	4.4 ^#^	210 ^†^	0.2 ^§^	n.t.	n.t.	3.0 ^†^	2800 ^†^	0.60/9.0 ^†^	13.7 ± 1.1	9.1 ^†^	[49,55,57,60]
Ziprasidone	9 *	2.7 **	1.5 ***	8 ^#^	n.t.	3 ^§^	n.t.	n.t.	n.t.	n.t.	n.t.	n.t.	n.t.	[49,63]

Abbreviations: 5-HT = serotonin; D = dopamine; M = muscarinic; α = alpha-adrenergic; n.t. = not tested; IC50 = half maximal inhibitory concentration. [3 H]ligand used Kd of ligand, nM: * = spiperone; ** = raclopride (1.9); *** = raclopride (1.6); ^#^ = spiperone (0.086); ^§^ = ketanserin; ^†^ = In vitro binding affinities for human receptors; ^$^ = rat striatum; ^£^ = rat total cortex.

## Data Availability

Raw data extracted from included studies, raw data used for analyses, and any other unpublished materials used in the review is freely available upon request to the corresponding author.
